# Transcriptome and Metabolome Profiling Provide Insights into Flavonoid Biosynthesis and the Mechanism of Color Formation in *Zanthoxylum bungeanum* Maxim.

**DOI:** 10.3390/plants14040558

**Published:** 2025-02-12

**Authors:** Lin Yang, Lu Tian, Jingwei Shi, Anzhi Wei

**Affiliations:** 1 College of Forestry, Northwest A&F University, Yangling 712100, China; yanglingem@163.com (L.Y.); t1anlu@nwafu.edu.cn (L.T.); 2 State Key Laboratory of Soil Erosion and Dryland Farming on the Loess Plateau, Institute of Soil and Water Conservation, Chinese Academy of Sciences and Ministry of Water Resources, Yangling 712100, China

**Keywords:** *Zanthoxylum bungeanum* Maxim., transcriptome, metabolome, flavonoid, coloring mechanism, development stages

## Abstract

The color of *Zanthoxylum bungeanum* Maxim. (*Z. bungeanum*) is a key quality indicator and a factor limiting the development of its industry. However, the underlying mechanisms governing color formation remain largely unexplored. In this study, an integrative analysis of transcriptome and metabolome profiles was conducted across four developmental stages to elucidate the color formation mechanism in *Z. bungeanum*. A total of 137 flavonoids were identified as the fruits ripened, with high levels of differentially accumulated metabolites (DAMs), including tricetin and (-)-epigallocatechin, which were strongly associated with color formation. This suggests their significant contribution to the pigmentation process. Nine differentially expressed genes (DEGs) were identified as candidate genes involved in color development. Additionally, 15 transcription factors (TFs) (12 MYB and 3 bHLH) exhibited expression patterns similar to those of structural genes in the flavonoid biosynthetic pathway, indicating their role in regulating flavonoid synthesis. The bioinformatics analysis of three key flavonoid synthesis genes—*ZbCHI*, *ZbFLS*, and *ZbANR*—revealed that all three proteins exhibit hydrophobic structures without transmembrane domains. Among them, *ZbANR* possesses signal peptide regions, whereas *ZbCHI* and *ZbFLS* do not. Subcellular localization predictions suggest that *ZbCHI* is most likely localized in the chloroplast, *ZbFLS* in the cytoplasm, and *ZbANR* in the membrane. Functional analyses revealed that their transient expression in *Nicotiana benthamiana* (*N. benthamiana*) increased the flavonoid content, with *ZbANR* overexpression producing a distinct white phenotype in the plants. This study enriches transcriptomic data and provides a comprehensive understanding of flavonoid metabolism and the molecular basis of color formation in *Z. bungeanum*, offering a valuable theoretical foundation for future breeding programs.

## 1. Introduction

Flavonoids, as a large family of secondary metabolites, are widely distributed in angiosperms [1]. More than 9000 flavonoids have been identified, including flavonols, flavones, flavanones, flavan-3-ols, isoflavones, and anthocyanins [2]. Flavonoids play an important role in plant pigment accumulation and the communication of environmental signals, and can act as antioxidants or signaling molecules to protect plants from the surrounding environment [3]. In addition, they also play a key role in medical health. Flavonoids are an indispensable ingredient in a healthy diet for humans and can improve cardiovascular diseases and prevent cancer [4]. In addition, they can also be effective in anti-inflammatory and bacteriostatic applications and can improve visual function [5].

The biosynthesis of flavonoids is highly complex, involving the regulation of internal factors such as biosynthetic enzymes, hormones, and transcription factors (TFs), as well as environmental factors like the temperature and nutrient availability [6,7]. The flavonoid biosynthetic pathway in plants has been extensively elucidated. Flavonoids are synthesized from phenylalanine via the phenylpropanoid and flavonoid pathways [8]. The process begins with the condensation of malonyl-CoA and 4-coumaroyl-CoA to form chalcones, a reaction catalyzed by chalcone synthase (*CHS*) [9]. *CHS* then catalyzes the production of naringenin chalcone, which is converted into naringenin by chalcone isomerase (*CHI*), providing the precursor for various flavonoid derivatives [10]. Finally, a series of key downstream enzymes, including *DFR* (dihydroflavonol-4-reductase), *FLS* (flavonol synthase), *LAR* (leucoanthocyanidin reductase), *LDOX* (leucoanthocyanidin dioxygenase), *ANR* (anthocyanidin reductase), and *UFGT* (UDP-glucose: flavonoid 3-O-glucosyltransferase), catalyze the formation of end products such as anthocyanins, isoflavones, flavonols, and proanthocyanidins (PAs) [2].

The flavonoid metabolic pathway regulates downstream reactions and end products through key rate-limiting enzymes, playing a decisive role in flavonoid biosynthesis [2]. Among these enzymes, *CHI* is crucial, catalyzing the conversion of naringenin chalcone into naringenin. *CHI* has been identified in various plants, including *Ginkgo biloba* [11], *Ipomoea batatas* [12], *Paeonia lactiflora* [13], and *Glycine max* [14]. Studies suggest that *CHI* increases the content of flavonols and flavones while reducing anthocyanin levels [15]. Flavonols, important pigments that determine petal color, are synthesized by *FLS*, which catalyzes the conversion of dihydroflavonols to flavonols [16]. Research shows that *FLS* affects flower color; the transient expression of *CnFLS1* in *Nicotiana benthamiana* (*N. benthamiana*) changed the flower color to white [16]. *FLS* and *DFR* work synergistically to regulate the balance between the anthocyanin and flavonol branches of the flavonoid pathway, ultimately influencing flower color formation [17]. The last step before the final divergence between epicatechin and anthocyanin synthesis is catalyzed by anthocyanidin synthase (*ANS*), which controls anthocyanin formation [2]. In *Rhododendron*, transient expression experiments in *N. benthamiana* revealed that *ANS* significantly enhances anthocyanin synthesis [18]. While these genes have been studied in many plants, reports in the Rutaceae family, except for *citrus*, are rare.

**Figure 1:**
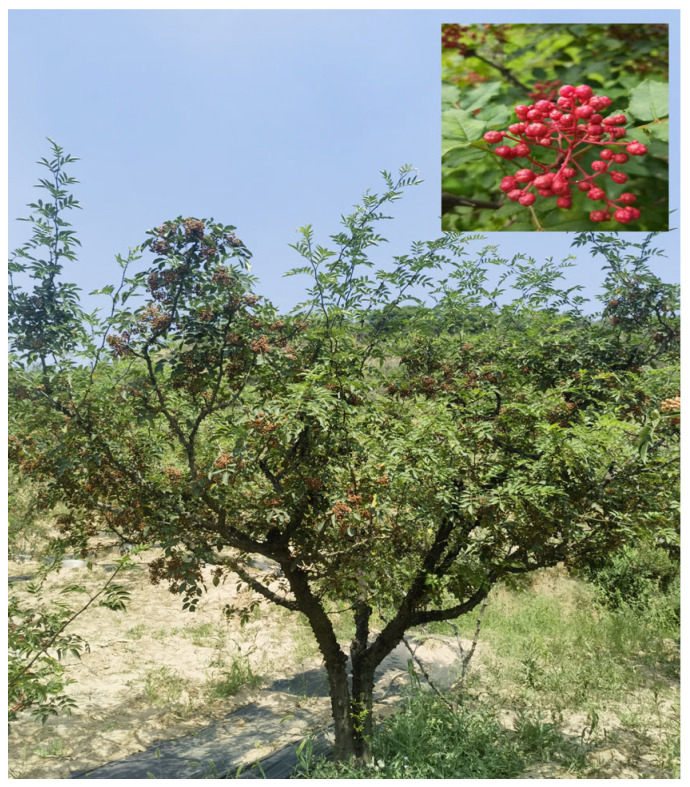
The whole tree and fruit features of *Z. bungeanum*.

An integrative analysis of the transcriptome and metabolome is an effective approach for identifying genes associated with the phenotype of interest and gaining insights into gene-to-metabolite networks [19,20]. In recent years, this approach has uncovered the molecular regulatory mechanisms of flavonoid biosynthesis in various plants. For example, one study identified 222 differentially accumulated metabolites (DAMs) and 15,855 differentially expressed genes (DEGs) in apricot fruit, providing a scientific basis for flavonoid biosynthesis in apricots [21]. Through transcriptomic and metabolomic analyses, 441 DAMs and 75 genes involved in the flavonoid biosynthesis pathway were identified in sea buckthorn, and the overexpression of *HrMYB114* significantly increased the flavonoid content in sea buckthorn [22]. The integrative analysis of metabolomics and transcriptomics also revealed the color formation mechanism in pepper fruits, showing that most flavonoids were highly accumulated in purple peppers [23]. Thus, integrating metabolite and gene expression profiles enables the identification of key gene networks regulating flavonoid biosynthesis during fruit peel development. *Zanthoxylum bungeanum* Maxim. (*Z. bungeanum*), also known as Chinese red pepper (Figure 1), belongs to the family Rutaceae [24,25]. As a medicinal and edible plant, it is widely used and holds great potential. Recent studies have reported that *Z. bungeanum* exhibits inhibitory effects on cancer cells and has potential as a biofuel source [26,27]. The fruit development of *Z. bungeanum* involves a color transition from cyan to bright red, where color serves not only as an indicator of ripening but also as a key quality evaluation parameter. Unfortunately, research on *Z. bungeanum* remains limited, with most studies focusing on its chemical components [28,29]. Flavonoids in *Z. bungeanum* have garnered significant attention due to their abundance and diverse bioactivities [30,31]; however, our understanding of them remains limited.

Based on this, flavonoids have garnered our attention, and we hypothesize that they may play a key role in the color development of *Z. bungeanum* fruit. To investigate the color formation mechanism in *Z. bungeanum*, we used liquid chromatography–tandem mass spectrometry (https://www.sciencedirect.com/topics/agricultural-and-biological-sciences/liquid-chromatography-mass-spectrometry, accessed on 12 June 2020) (LC–MS/MS) to detect and quantify flavonoids in *Z. bungeanum* fruits at four developmental stages. Based on transcriptomics, the key genes that related to flavonoid synthesis were screened out. The accuracy of the selection was confirmed using qRT-PCR. In addition, bioinformatics analysis and heterologous transient transformation were used to further validate the function of key genes. The findings from this study will enrich the transcriptomic data and contribute to the development of high-flavonoid varieties through breeding.

## 2. Results

### 2.1. Metabolic Differences in the Fruits of Z. bungeanum

**Figure 2:**
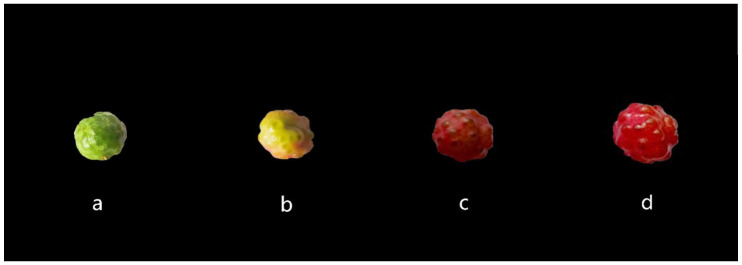
*Z. bungeanum* fruits from 4 development stages. (**a**) First stage (nondiscoloration period); (**b**) second stage (early discoloration period); (**c**) third stage (mid-discoloration period); (**d**) fourth stage (complete discoloration period).

**Figure 3:**
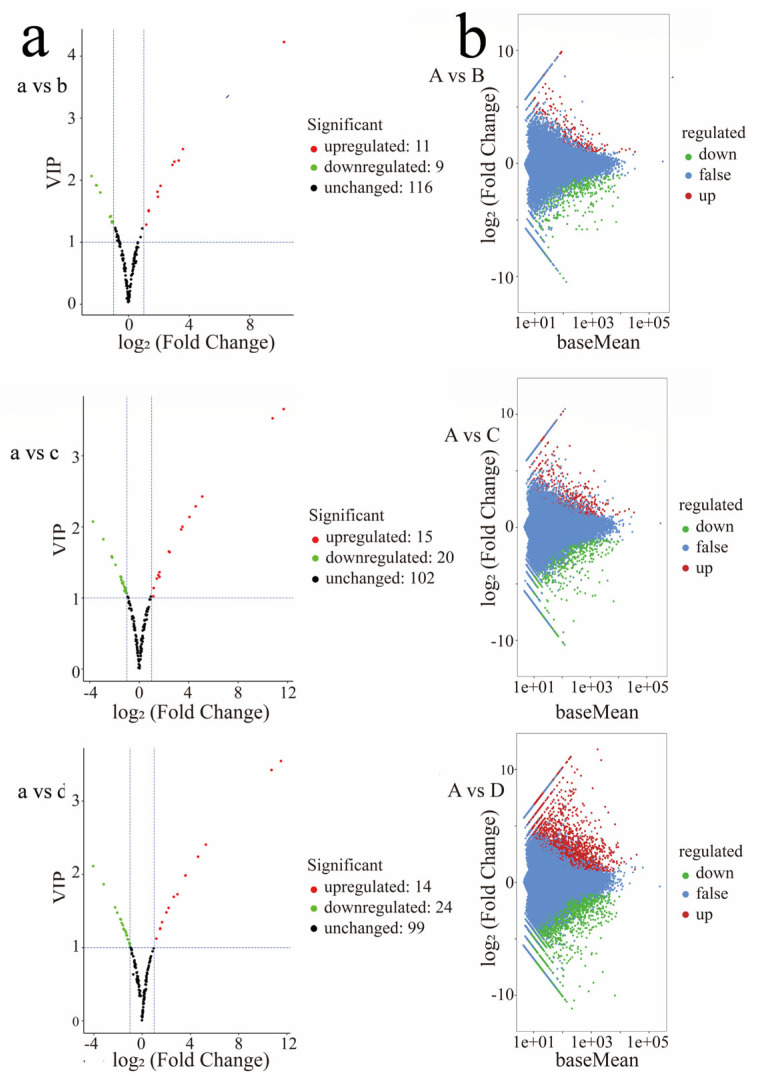
Analysis of DAMs and DEGs. (**a**) Volcano plots of DAMs. Each point in the map represents a metabolite, and the abscissa represents the logarithm of the quantitative difference multiples of a metabolite in the two samples. The ordinate represents the VIP value. Green dots represent downregulated metabolites, red dots represent upregulated metabolites, and black dots represent metabolites detected but not significantly different. (**b**) MA map of DEGs. The ordinate represents the log_2_Fold change. The abscissa represents the mean value of gene expression in two samples. Red dots represented upregulated gene expression, green dots represented downregulated gene expression, and blue indicates no significant difference in gene expression.

To understand the molecular mechanisms underlying color in *Z. bungeanum*, fruit samples from four developmental stages (Figure 2) were analyzed using liquid chromatography–tandem mass spectrometry (LC-MS/MS). The four developmental stages were labeled as a, b, c, and d in chronological order. A total of one hundred and thirty-seven flavonoids were detected across these stages, including thirty-five flavonoids, thirteen dihydroflavones, eleven proanthocyanidins, nine flavonoid carbonosides, eight dihydroflavonols, seven flavanols, six anthocyanins, and two chalcones (Table S1). A fold change of ≥ 2 or ≤ 0.5, along with a VIP (variable important in projection) value of ≥ 1, was set as the threshold for identifying DAMs. Based on these criteria, the number of DAMs ranged from 2 to 38 across the four developmental stages (Figure 3a). The number of DAMs was highest during the a-b period, with 11 metabolites upregulated and 9 downregulated. Most of the detected metabolites were flavonoids and anthocyanins, marking this period as crucial for the fruit’s transition from green to red. Therefore, the a-b period was a key stage for color accumulation in *Z. bungeanum* fruits. During this time, a large number of flavonoid DAMs were detected, which were positively correlated with fruit color development. After this stage, the color remained stable, and flavonoid levels decreased. Additionally, we believe that flavonoid accumulation in the fruit may follow a gradual decline throughout the developmental process.

The Kyoto Encyclopedia of Genes and Genomes (KEGG) database is a valuable resource for researchers, enabling the study of genes, expression data, and metabolite content within an integrated network. The KEGG annotation of significantly different metabolites revealed that most were concentrated in the flavonoid biosynthesis pathway (Figure S1). To further explore the relationship between fruit color in *Z. bungeanum* and flavonoids, we analyzed the metabolites that exhibited significant differences during the a-b period (Table 1). Among the metabolites, tricetin was the most significant DAM, with a pronounced upregulation during the a-b period. Tricetin is believed to play a crucial role in the transformation of *Z. bungeanum* fruit from green to red. Notably, tricetin was the most aggressively upregulated across all developmental stages. Additionally, tricetin is located in the flavonoid biosynthesis pathway (KEGG, ko00941). Most of the upregulated metabolites were flavonoids and anthocyanins, indicating their close association with the accumulation of red color in the fruits. The downregulated metabolites included dihydroflavonols and flavanols, with (-)-epigallocatechin being the most significantly downregulated metabolite across all developmental stages, which is believed to be linked to the fruit’s green characteristics.

### 2.2. Transcriptome Analysis

In this experiment, transcriptomic sequencing was conducted on 12 fruit samples from four developmental stages, designated A, B, C, and D in chronological order. A total of 82.32 GB of clean data was obtained, with each sample yielding at least 6 GB of clean data. The Q30 base percentage exceeded 90% (Table S2). The transcript sequences were obtained using Trinity, and the longest cluster sequences generated from Corset hierarchical clustering were designated as unigenes for subsequent analysis. A total of 327,752 unigenes were identified, with an average length of 889 nt and an N50 length of 1228 nt (Table S3). These results indicated that the transcriptome data were of high quality and suitable for further study.

The basic local alignment search tool (BLAST) was used to compare the unigene sequences against the KEGG, NR (NCBI non-redundant protein sequences), Swiss-Prot (a manually annotated and reviewed protein sequence database), GO (Gene Ontology), and COG/KOG databases (Clusters of Orthologous Groups of proteins). After predicting the amino acid sequences of the unigenes, HMMER software (version 3.3) was utilized to compare them with the Pfam database (protein family), yielding annotation information for the unigenes. The annotation results from each database are presented in Table S4.

### 2.3. DEGs in Fruits of Different Development Stages

For samples with biological replicates, DESeq2 is suitable for differential expression analysis between sample groups, allowing for the identification of differentially expressed gene sets across two biological conditions [32]. DEGs were identified using DESeq, resulting in the following annotations: 1597 DEGs (1004 upregulated and 593 downregulated) for the comparison of A vs. B; 2944 DEGs (1735 upregulated and 1209 downregulated) for A vs. C; and 9772 DEGs (5010 upregulated and 4762 downregulated) for A vs. D (Figure 3b).

GO annotation, which is applicable to a variety of species, helps define and describe gene and protein functions. The DEGs were annotated in GO and were found to be involved in a “metabolic process”, “cellular process”, “cell”, “cell part”, “catalytic activity”, and “binding” (Figure S2). These DEGs offer insights into the molecular mechanisms underlying fruit color in *Z. bungeanum*. In organisms, different gene products perform biological functions through interactions, and the pathway annotation analysis of DEGs helps us further interpret gene functions. KEGG analysis was performed on the DEGs. A total of 715 (A vs. B), 1232 (A vs. C), 3843 (A vs. D), 508 (B vs. C), 2106 (B vs. D), and 1204 (C vs. D) DEGs were mapped to 129, 135, 143, 114, 137, and 122 KEGG pathways, respectively (Figure S3). KEGG annotations were primarily concentrated in metabolic and secondary metabolic pathways, with a significant portion of DEGs mapped to flavonoid biosynthesis, indicating that flavonoids play an important role in the fruit development of *Z. bungeanum* (Figure S4).

**Figure 4:**
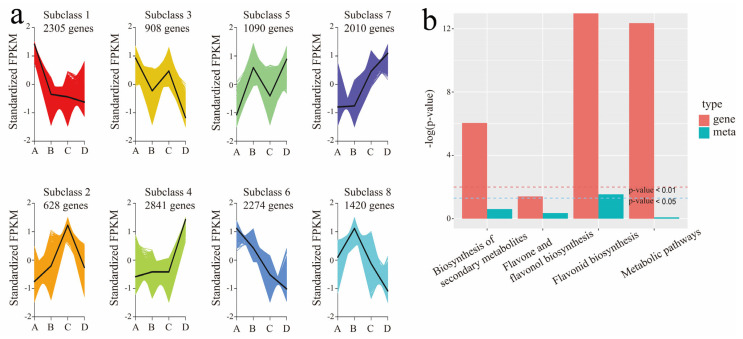
Integrated analysis of transcriptome and metabolome: (**a**) K-means cluster analysis, co-expressed genes during fruit ripening. The x-coordinate represents the sample, and the y-coordinate represents the centralized and standardized expression. (**b**) KEGG enrichment analysis. The horizontal axis represents the metabolic pathway, the vertical axis represents the enrichment *p*-value of differential genes, and the blue represents the enrichment of differential metabolites.

Cluster analysis is useful for assessing gene expression patterns under different experimental conditions. By grouping genes with similar expression patterns, it becomes possible to infer the functions of unknown genes or uncover unknown functions of known genes. To further understand the dynamics of gene expression changes during fruit development, the fragments per kilobase of exon model per million mapped fragments (FPKM) values of genes were centralized and standardized, followed by K-means cluster analysis. This resulted in eight distinct clusters of co-expressed genes with significant transcriptional bursts, which are essential for the transition from green to fully red in *Z. bungeanum* fruits (Figure 4). A total of 2305, 905, and 2275 genes were clustered in clusters 1, 3, and 6, respectively. These clusters displayed a decrease in expression levels from the green to the fully red period, indicating a negative association with red coloration in the fruits. Notably, the expression levels of these genes dropped significantly during the critical a-b period, suggesting that silencing these genes is essential for the coloration of *Z. bungeanum* fruits. In clusters 4 and 7, with 2841 and 2010 genes, respectively, expression levels remained relatively stable during the a-b period, suggesting they are not involved in the fruit coloration process. In contrast, genes in clusters 2 (628 genes) and 5 (1090 genes) showed a positive association with red coloration as the fruit ripened, with expression levels upregulated. These clusters contain numerous transcription factors (TFs), including MYB and bHLH, which play a crucial role in regulating the transcription of genes related to fruit color. Interestingly, 1420 genes in cluster 8 exhibited a sharp upregulation during the a-b period, followed by rapid downregulation, resulting in a final expression level lower than the initial one, suggesting these genes may play a focused role in fruit coloring.

### 2.4. Genes Associated with Color Accumulation

**Figure 5:**
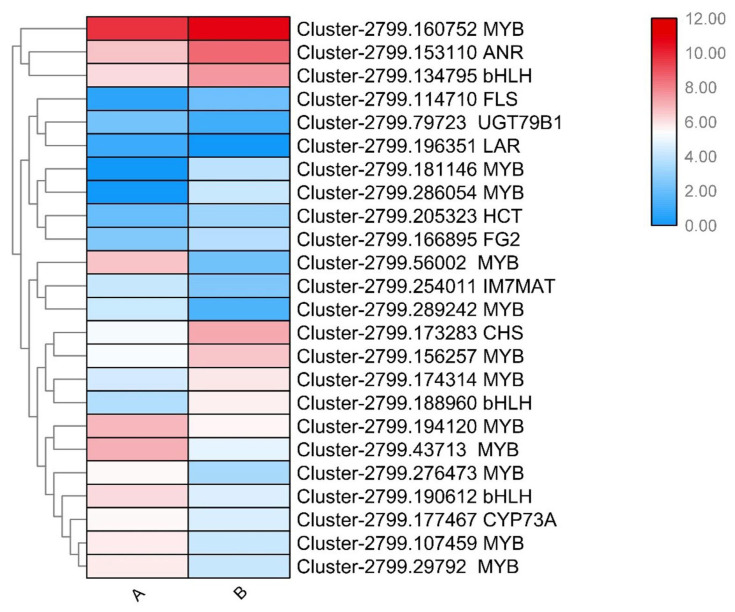
The heatmap of the expression pattern of the 9 color-related DEGs and 15 TFs (12 MYBs, 3 bHLHs). Note: A: first development stage of *Z. bungeanum* fruits; B: second development stage.

The three primary biosynthesis pathways linked to color accumulation are flavonoid biosynthesis, anthocyanin biosynthesis, and flavone and flavonol biosynthesis. The KEGG pathway enrichment analysis of all DEGs (A vs. B) identified 34 DEGs associated with these pathways. Of these, 30 were annotated to the flavonoid biosynthesis pathway, with 9 showing significant expression changes, suggesting that *FLS*, *CHS*, *ANR*, *HCT*, and *FG2* may contribute to red color accumulation in *Z. bungeanum* (Figure 5). Meanwhile, *CYP73A*, *UGT79B1*, *IF7MAT*, and *LAR* may play a role in the loss of green coloration in the fruits.

Gene expression in the flavonoid metabolic pathway is influenced not only by specific genes but also by TFs, such as MYB and bHLH [33]. In this study, we identified 105 differentially expressed TFs, including 12 MYB TFs and 3 bHLH TFs. A heatmap was constructed to elucidate the relationship between the DEGs and the differentially expressed TFs (Figure 5). To further elucidate the relationship between DEGs and TFs, correlation analysis was conducted. Some DEGs exhibited expression patterns that were consistent with those of TFs, such as Cluster-2799.160752 (*MYB*) and Cluster-2799.153110 (*ANR*), Cluster-2799.134795 (*bHLH*) and Cluster-2799.114710 (*FLS*), and Cluster-2799.56002 (*MYB*) and Cluster-2799.254011 (*IF7MAT*). By screening for correlations with a Pearson correlation coefficient (PCC) value greater than 0.8, we identified significant relationships between the expression of Cluster-2799.173283 (*CHS*) and Cluster-2799.174314 (*MYB*), Cluster-2799.205323 (*HCT*) and Cluster-2799.188960 (*bHLH*), and Cluster-2799.79723 (*UGT79B1*) and Cluster-2799.276473 (*MYB*), as well as Cluster-2799.254011 (*IF7MAT*) and Cluster-2799.56002 (*MYB*). Conversely, some genes and TFs exhibited a negative correlation, with PCC values less than -0.8, including Cluster-2799.177467 (*CYP73A*) and Cluster-2799.156257 (*MYB*), Cluster-2799.79723 (*UGT79B1*) and Cluster-2799.174314 (*MYB*), Cluster-2799.153110 (*ANR*) and Cluster-2799.276473 (MYB), and Cluster-2799.166895 (*FG2*) and Cluster-2799.276473 (*MYB*).

### 2.5. Integrated Analysis of Flavonoid Biosynthesis in Z. bungeanum

The integration of multiple omics analyses can address data issues arising from data loss and other factors. Additionally, the mutual validation among various omics can help reduce false positives associated with single-omics analyses. In this experiment, we selected key genes and metabolites through integrated analysis for subsequent in-depth experimental investigation and application. The integrated analysis groups were designated as I (A vs. a), J (B vs. b), K (C vs. c), and L (D vs. d).

Based on the results of the enrichment analysis of DAMs and DEGs, a histogram was created to illustrate the enrichment levels of pathways containing both DAMs and DEGs. Our findings indicated that both DEGs and DAMs exhibited the highest enrichment in the flavonoid biosynthesis pathway from the I vs. J comparison (Figure 4b), prompting us to focus on the I vs. J period (a–b).

According to the DAM analysis conducted in this experiment, along with the DEG analysis, we simultaneously mapped the DEGs and DAMs within the same group to the flavonoid biosynthesis pathway to enhance our understanding of the relationship between genes and metabolites. A total of 30 DEGs (6 upregulated and 8 downregulated) and 8 DAMs were annotated within the flavonoid biosynthesis pathway.

**Figure 6:**
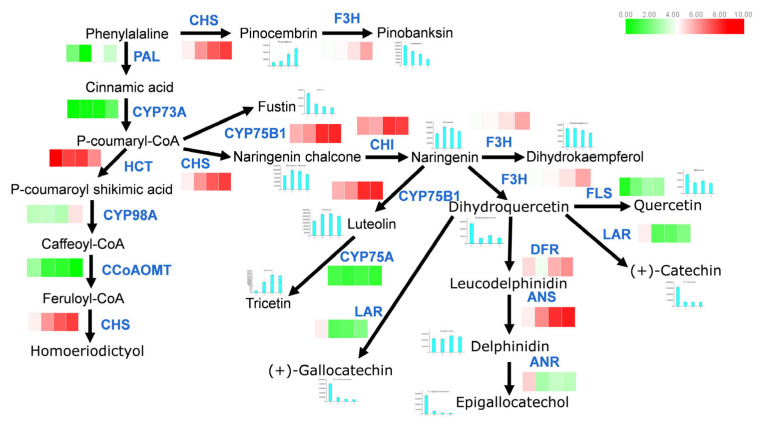
Regulation of flavonoid biosynthesis pathway during *Z. bungeanum* ripening. This pathway was constructed based on the KEGG annotation results. Blue letters indicate structural genes; heatmap color blocks represent gene expression patterns (FPKM value). Black letters represent metabolites; the histogram displays changes in the main metabolites (relative content) during ripening. Gene abbreviations are as follows: *CYP73A* (trans-cinnamate 4-monooxygenase), *CHI* (chalcone isomease), *CYP75B1* (flavonoid 3′-monooxygenase), *DFR* (flavanone 4-reductase), *F3H* (flavonoid 3-hydroxylase), *HCT* (shikimate O-hydroxycinnamoyltransferase), *CYP98A* (coumaroylquinate (coumaroylshikimate) 3′-monooxygenase), *CCoAOMT* (caffeoyl-CoA O-methyltransferase), *CHS* (chalcone synthase), *PGT1* (phlorizin synthase), *ANS* (anthocyanidin synthase), *FLS* (flavonol synthese), *LAR* (leucoanthocyanidin reductase), *ANR* (anthocyanidin reductase), *PAL* (phenylalanine ammonia-lyase), *CYP75A* (flavonoid 3′, 5′-hydroxylase).

Transcriptome and metabolome analyses highlighted flavonoid biosynthesis-related DEGs and DAMs, which may underlie the changes in the fruit coloration of *Z. bungeanum*. Based on the analysis results, a brief flavonoid biosynthesis pathway for *Z. bungeanum* was constructed (Figure 6 and Figure S5).

Generally, two classes of genes are involved in flavonoid synthesis during the development of *Z. bungeanum*. The first group of genes exhibited high activity at all developmental stages, including *CHS*, *HCT*, *CYP75B1*, *F3H*, *CHI*, *DFR*, and *ANS*. Among these, *ANS*, *CHS*, *CYP75B1*, and *HCT* showed particularly high activity during the a-b period, suggesting a correlation with the reddening of the fruits.

The second group of genes displayed lower activity throughout all developmental stages, with *CYP73A* and *CYP75A* being completely silenced during all phases. Notably, some metabolites, such as tricetin, luteolin, naringenin, and naringenin chalcone, significantly increased during the a-b period, indicating their potential role in the accumulation of red pigments.

### 2.6. Quantitative Real-Time PCR (qRT-PCR) Verification of RNA Sequencing (RNA-Seq) Results

To verify the accuracy of the RNA-seq data, 10 DEGs were selected (Table S5). qRT-PCR was conducted to analyze the expression levels of these genes across the four developmental stages (Figure S6). The results demonstrated that the expression patterns observed in qRT-PCR were consistent with the RNA-seq findings, indicating that the expression data obtained from RNA-seq are valid and reliable.

### 2.7. Molecular Cloning and Bioinformatics Analysis

Naringenin is a core intermediate in flavonoid biosynthesis, and naringenin chalcone is catalyzed to synthesize naringenin by *CHI*, which is considered a key gene in the flavonoid pathway [23]. In this study, we selected two candidate genes related to color accumulation (*FLS* and *ANR*) along with *CHI* for further investigation. The coding sequences (CDSs) of *ZbCHI*, *ZbFLS*, and *ZbANR* were cloned. The *ZbCHI* CDS (MT731945) is 1248 base pairs (bp) in length and encodes 416 amino acids, while that of *ZbFLS* (MT731946) is 903 bp and encodes 301 amino acids, and that of *ZbANR* (MT731947) is 870 bp, encoding 290 amino acids. Three fragments were amplified using real-time PCR, yielding results consistent with the expected sizes (Figure S7).

The analysis of the deduced amino acid sequences of these three genes was performed using the BLAST, revealing a high degree of homology for *ZbCHI* with other plant species, such as *Citrus clementina* (KDO73652.1, 88.14% identity) and *Citrus sinensis* (KDO73655.1, 81.92% identity) (Figure S8). *ZbFLS* exhibited 80.23% and 78.71% identity with *FLS* from *Citrus sinensis* (XP_006466183.1) and *Pistacia vera* (XP_031286995.1) (Figure S9). For *ZbANR*, it showed 87.2% and 82.35% identity with *ANR* from *Citrus clementina* (XP_006449144.1) and *Pistacia vera* (XM_031391609.1) (Figure S10).

To understand the characteristics of the genes and provide a reference for further study, we performed bioinformatics analysis on the sequences. The predictions of the hydrophilicity and hydrophobicity of the proteins revealed that all three proteins exhibited hydrophobic structures (Figure S11). TMHMM analysis indicated that none of the three proteins contained transmembrane domains (Figure S12). Results from the SignalP 3.0 Server indicated that *ZbANR* possessed signal peptide regions, while *ZbCHI* and *ZbFLS* did not. Subcellular localization predictions showed that *ZbCHI* had a higher probability of being localized to chloroplasts, *ZbFLS* was more likely to be found in the cytoplasm, and *ZbANR* had a higher probability of being associated with membranes (Figure S13).

### 2.8. The Overexpression of ZbCHI, ZbANR, and ZbFLS Alters the Phenotype of N. benthamiana

**Figure 7:**
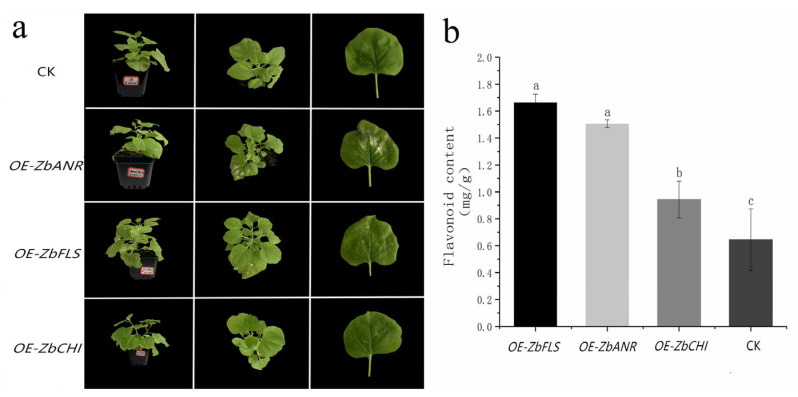
Transient expression of *ZbFLS*, *ZbANS*, and *ZbCHI* in *N. benthamiana* enables flavonoid production. (**a**) Infiltration of *Agrobacteria*-harboring plasmids for the expression of *ZbANR* and *ZbFLS* in *N. benthamiana* leaves causes white pigmentation in the infiltrated area; (**b**) detection of the flavonoid content in *N. benthamiana*. Values indicate means of three biological replicates ± SD. The letters indicate differences in the flavonoid content among *N. benthamiana* plants with different transient overexpression treatments according to Duncan’s multiple range test.

Compared with model plants such as *rice* and *Arabidopsis thaliana*, the genetic transformation system of *Z. bungeanum* is challenging due to its long growth cycle. Transient expression is an effective method for functional research in such cases. To further investigate the roles of *ZbCHI*, *ZbFLS*, and *ZbANR*, transient overexpression was conducted in *N. benthamiana* leaves. CDSs were cloned into the pBI121 vector, and each gene was expressed individually in *N. benthamiana*. The empty vector was used as a control. After eight days of *Agrobacterium*-mediated transformation, phenotypic changes were observed, and the flavonoid content was measured (Figure 7).

Compared with the control, the overexpression of both *ZbANR* and *ZbFLS* induced a white pigmentation phenotype in the *N. benthamiana* leaves, with *ZbANR* overexpression showing a more pronounced effect on white pigment precipitation. In contrast, the overexpression of *ZbCHI* had no significant impact on the phenotype. Flavonoid content analysis revealed that the overexpression of all three genes significantly increased the flavonoid content in *N. benthamiana* compared with the control, with *ZbFLS* showing the most substantial effect in promoting flavonoid accumulation (a 2.54-fold difference). These results suggested that the overexpression of *ZbFLS*, *ZbANS*, and *ZbCHI* could promote flavonoid synthesis and accumulation, leading to the difference in the *Z. bungeanum* peel color.

## 3. Discussion

The bright red color of ripe *Z. bungeanum* is a key quality indicator [26]. However, post-harvest, the color tends to darken or become black-red due to environmental factors, affecting its quality [30]. Despite the importance of color, the molecular mechanisms underlying color formation in *Z. bungeanum* are not well understood. Transcriptomics integrated with metabolomics offers valuable insights for identifying genes associated with color accumulation [30,34]. In this study, we analyzed four developmental stages of *Z. bungeanum* fruit using transcriptomic and metabolomic approaches, shedding light on previously unresolved questions.

Flavonoids are significant contributors to flower color in ornamental plants, producing pigments that create pink, red, orange, and blue hues [1]. These pigments are also abundant in *Zanthoxylum bungeanum*, drawing our research interest. In this study, a widely targeted metabolomics approach enabled the detection of 137 flavonoid metabolites, providing a comprehensive view of flavonoids involved in color changes during *Z. bungeanum* fruit development. The main flavonoids identified included flavonols, flavonoids, dihydroflavones, flavanols, dihydroflavonols, anthocyanins, proanthocyanidins, chalcones, and flavonoid carbonosides. Observing the phenotype, the a-b period was identified as the critical phase during which the fruit color shifts from green to red, aligning well with the metabolomic data. In this study, tricetin garnered significant attention as it was the most dramatically upregulated DAM throughout the developmental stages and is part of the flavonoid biosynthesis pathway. Tricetin, a flavonoid derivative also found in Myrtaceae pollen and *Eucalyptus* honey, is known for its strong anti-inflammatory and anti-cancer effects [35,36]. Tricetin is considered a significant flavonoid that plays a critical role in red pigment accumulation and contributes to the medicinal properties of *Z. bungeanum*, meriting further investigation [35]. Additionally, cyanidin 3-rutinoside and laricitrin-O-acetylhexoside were sharply upregulated during development, suggesting their involvement in red pigment accumulation in *Z. bungeanum* fruit. Notably, some DAMs, such as (-)-epigallocatechin and (+)-gallocatechin, were dramatically downregulated, potentially linking them to the loss of green pigment in the fruit [37]. Interestingly, few studies have explored the relationship between these flavonoids and plant coloration; we hope this study provides valuable insights into plant color mechanisms.

The DEG annotation results indicated that most DEGs were concentrated in the flavonoid biosynthesis pathway, underscoring the pivotal role of flavonoids in the development and coloration of *Z. bungeanum* [31]. KEGG enrichment results identified nine genes related to flavonoid biosynthesis during the a-b period, suggesting their potential involvement in color changes in *Z. bungeanum*. Among these, *FLS* was the most upregulated gene, utilizing dihydroflavonol as a substrate to produce colorless flavonols [2], with *FLS* expression directly influencing the flavonol content [38]. Flavonols can form a copigmentation with anthocyanins, leading to color shifts and enhancing color diversity [1,30]. The high expression of *FLS* may produce flavonols, contributing to color change in *Z. bungeanum* [30]. Given the significant role of *FLS*, its function was further characterized in *Z. bungeanum*. The transient expression of *ZbFLS* in *N. benthamiana* resulted in an increased flavonoid content and a slightly white phenotype, consistent with previous findings [16,39]. Among the candidate genes, *ANR* was notably upregulated. As a key downstream enzyme in the anthocyanin biosynthetic pathway—an important branch of the flavonoid pathway—*ANR* catalyzes the conversion of anthocyanins to catechin and epicatechin, which contribute to the red, purple, and blue pigmentation in many plants [1,2]. In *Z. bungeanum*, the transient expression of *ZbANR* significantly increased the flavonoid content and altered the phenotype of *N. benthamiana*, suggesting that *ZbANR* may play a particularly prominent role in coloration compared to other genes [40]. The relatively low expression of *LAR*, associated with low levels of epigallocatechin, aligns with metabolome results and previous findings [41], indicating that *LAR* may be a key gene in the disappearance of green pigmentation in *Z. bungeanum*.

The flavonoid biosynthesis pathway is regulated by structural genes [42]. In this study, we mapped the flavonoid regulatory network of *Z. bungeanum*, categorizing the genes into two groups: upregulated and downregulated. Interestingly, certain genes displayed unique expression patterns. Notably, the expression level of *DFR* decreased rapidly during the fruit coloring stage, then steadily increased during the red pigment accumulation phase. This suggests potential competition between *DFR* and *FLS* during the a-b period, where *FLS* expression was ultimately suppressed, inhibiting flavonol synthesis and favoring anthocyanin production [43]. This shift likely plays a key role in the reddening of *Z. bungeanum* fruits in the later stages of development [44]. Furthermore, the sustained high expression of *ANS* in the downstream pathway influenced delphinidin synthesis [45]. Among the six common anthocyanidins in plants, delphinidin has been reported to contribute to red coloration in many species [46]. We therefore suggest that delphinidin is a key anthocyanin associated with the red pigmentation in *Z. bungeanum*.

Previous reports indicated that structural genes in the flavonoid biosynthesis pathway are regulated by TFs, such as MYB TFs [6,34,39]. MYB TFs are believed to be involved in regulating flavonoid synthesis in *Z. bungeanum* [30]. In this experiment, we focused on the expression of MYB TFs. Certain MYB TFs showed expression patterns similar to those of structural genes during the a-b period, including Cluster-2799.160752 MYB with Cluster-2799.153110 ANR, Cluster-2799.156257 MYB with Cluster-2799.173283 CHS, and Cluster-2799.107459 MYB with Cluster-2799.177467 CYP73A, implying that these MYB TFs may positively regulate these genes. Additionally, some bHLH and MYB TFs displayed similar expression patterns, suggesting potential interactions that may jointly regulate the flavonoid biosynthesis pathway and stamen development in *Z. bungeanum*, aligning with previous research [47,48]. However, the specific mechanisms in *Z. bungeanum* remain unclear and require further investigation.

## 4. Materials and Methods

### 4.1. Plant Material

In this study, we selected *Zanthoxylum bungeanum* CV. ‘Fengxiandahongpao’, the most representative variety, as the plant material. The fruits of *Z. bungeanum* were divided into four developmental stages from green to completely red according to the maturity of the fruits (Figure 2). The colors of *Z. bungeanum* peels at those four stages, including the nondiscoloration period, early discoloration period, mid-discoloration period and complete discoloration period, are significantly different. Thus, samples from four developmental stages of *Z. bungeanum* fruits were collected for transcriptomic and metabolomic analyses. These samples were obtained from a common garden in a semi-arid area on the Loess Plateau (Sassafras Engineering Technology Research Center of the State Forestry Administration, Fengxian County, Shaanxi Province, China; 33°59′ N, 106°39′ E) [24,49,50]. All trees were of the same age (8 years). At each stage, with three randomly selected trees used as biological replicates, 300–400 fruits were collected from each tree. All fruits were uniform in color without signs of mechanical damage or disease. Samples from each developmental stage were divided into three parts: one for transcriptome analysis, one for metabolomic analysis, and one for RT-qPCR. During sampling, fruits were promptly peeled, and peels were flash-frozen in liquid nitrogen and stored at −80 °C until further use.

### 4.2. RNA Extraction, Library Construction, RNA-Seq, and Assembly

A total of 12 sample groups from four developmental stages were used for RNA extraction. The total RNA of fruit peel samples was extracted using the RNAprep Pure Plant kit (Tiangen, Beijing, China). The RNA integrity and concentration were measured using an Agilent 2100 Bioanalyzer (Agilent Technologies, Inc., Santa Clara, CA, USA). The mRNA was isolated using the NEBNext Poly (A) mRNA Magnetic Isolation Module (NEB, E7490). The cDNA library was constructed using the NEBNext Ultra RNA Library Prep Kit for Illumina (NEB, E7530) and NEBNext Multiplex Oligos for Illumina (NEB, E7500) per the manufacturer’s instructions. A cDNA library for each sample was constructed and sequenced using the Illumina HiSeq4000 platform (Illumina Inc., San Diego, CA, USA). After filtering the raw sequencing data, high-quality reads were obtained, and the species’ transcriptome was assembled. Trinity software (version 2.0.6) (http://trinityrnaseq.sourceforge.net/, accessed on 12 June 2020) was used for clean read assembly, and the assembled transcript sequences served as the reference sequence. The longest cluster sequence obtained from Corset hierarchical clustering was designated as the unigene for subsequent analyses. The FPKM (fragments per kilobase of transcript per million fragments) value was used to measure the transcript or gene expression levels. Based on raw count data, the DESeq2 R package (1.16.1) was applied for differential expression analysis between sample groups [32], with screening criteria for DEGs set at |log_2_Fold Change| ≥ 1 and a False Discovery Rate (FDR) < 0.05. Gene functions were annotated using seven databases: GO (Gene Ontology), KO (the Kyoto Encyclopedia of Genes and Genomes Ortholog database), KOG/COG (Clusters of Orthologous Groups of proteins), Nr (NCBI non-redundant protein sequences), Nt (NCBI non-redundant nucleotide sequences), Pfam (protein family), and Swiss-Prot (a manually annotated and reviewed protein sequence database). The transcripts’ raw data were deposited in the NCBI SRA database under project number PRJNA1195223.

### 4.3. Metabolome Analysis

Twelve *Z. bungeanum* fruit samples, representing four developmental stages, were collected for metabolite extraction. The fruit peel samples were vacuum freeze-dried in a freeze drier (Scientz-100F, Ningbo, China) and then ground into powder using a grinding mill (MM 400, Retsch, Germany). A total of 300 mg powder was dissolved in 1.2 mL 70% aqueous methanol (*v*/*v*) and mixed well with a Vortex-6 (Kylin-Bell, Haimen, China). The homogenate was extracted overnight at 4 °C and then centrifuged (5424R, Eppendorf Co., Shanghai, China) at 10,000 rpm at 4 °C for 10 min, and the supernatant was collected. The supernatant was filtered using a microporous membrane filter (a 0.22 μm pore size) and stored in injection vials for analysis on an LC-ESI-MS/MS system (HPLC: Shim-pack UFLC SHIMADZU CBM30A system, www.shimadzu.com.cn/; MS: Applied Biosystems 4500 Q TRAP, https://sciex.com/products/mass-spectrometers/qtrap-systems/qtrap-4500-system, accessed on 12 June 2020) at Metware Bio-Tech Co. (Wuhan, China). Data were analyzed by Metware Biotechnology Co., Ltd. (Wuhan, China, http://www.metware.cn/), with three replicates performed for each sample.

Qualitative data analysis was performed based on secondary spectrum information from the MWDB database compiled by Metware Biotechnology Co., Ltd. (Wuhan, China). The identified metabolites were analyzed using orthogonal partial least squares discriminant analysis (OPLS-DA). Metabolites with a fold change of ≥2 or ≤0.5 and a variable importance in projection (VIP) score of ≥ 1 were classified as DAMs. The KEGG and the Plant Metabolic Network (PMN) databases were used to perform the pathway enrichment analysis of these flavonoids.

### 4.4. RT-qPCR Analysis

Total RNA was extracted from *Z. bungeanum* fruit at four developmental stages using the RNAprep Pure Plant Plus Kit (Tiangen, Beijing, China), following the manufacturer’s instructions. Reverse transcription was performed with reverse transcriptase (TransGen Biotech, Beijing, China) to synthesize cDNA. Ten DEGs were selected for RT-qPCR analysis, using UBA and TIF as reference genes for normalization [24]. Primers were designed with Primer Premier 5.0 (Palo Alto, CA, USA) and are listed in Table S5.

Gene expression profiles were examined using the Bio-Rad CFX96 Real-Time PCR system (Bio-Rad Laboratories Inc., Hercules, CA, USA), with each 25 μL reaction mixture containing 12.5 μL of TransStart Tip Green qPCR SuperMix (TransGen Biotech, Beijing, China), 9.5 μL ddH_2_O, 1 μL cDNA template, and 1 μL of each primer. The PCR program was initial denaturation at 94 °C for 30 s, followed by 45 cycles of 94 °C for 5 s and annealing at 60 °C for 30 s. The melting curve was recorded with steps at 95 °C for 5 s and 65 °C for 5 s, followed by continuous heating. Each reaction included three biological replicates, and the relative mRNA abundance was calculated using the 2^−ΔΔCt^ method.

### 4.5. Gene Cloning and Sequence Analysis

Full-length coding sequences (CDSs) of *ZbCHI*, *ZbFLS*, and *ZbANS* were cloned using primers listed in Table S5. Gene amplification was carried out using high-fidelity DNA polymerase (NEB, Beijing, China). The amplified target genes were cloned into the pC2300 control vector and subsequently transformed into *E. coli* TOP10 competent cells. Positive clones were selected and sequenced (Tsingke, Beijing, China). The obtained sequences were then compared with those of related species using BLASTX from the National Center for Biotechnology Information (NCBI) database to confirm the sequence integrity.

### 4.6. Plasmid Constructs

Based on the gene sequences and vector restriction sites, primers with homologous arms and restriction sites were designed to amplify the vector. The ClonExpress II One Step Cloning Kit (Vazyme, Nanjing, China) was used for ligation. To generate the constructs *pBI121-ZbCHI*, *pBI121-ZbFLS*, and *pBI121-ZbANS*, the coding sequence fragments were amplified using primers with compatible restriction sites and then cloned into the *XhoI* and *SalI* sites of the pBI121 vector.

### 4.7. Transient Expression Assay in *N. benthamiana*

Transient expression was conducted in *N. benthamiana* grown in a controlled greenhouse environment (28 ± 2 °C during the day, 25 ± 2 °C at night). The correctly sequenced transient expression vectors—*pBI121-ZbCHI*, *pBI121-ZbFLS*, *pBI121-ZbANS*, and an empty vector control (CK)—were transferred into *Agrobacterium tumefaciens* GV3101 competent cells (Biomed, Beijing, China), following the manufacturer’s protocol. Specifically, the transgenic bacterial solution was transferred to an LB liquid medium (containing kanamycin and rifampicin) and incubated until OD_600_ = 1.2. The transgenic bacterial solution was centrifuged at 6000× *g* at 18 °C for 10 min and the collected cells were resuspended in a resuspension solution (MS liquid medium) containing 40 mL 250 mM MES, 100 μL 10 mM MgCl_2_, and 3 mL 50 mM AS until OD_600_ = 0.6. *N. benthamiana* leaves growing vigorously in aseptic bottles were selected as infection objects. The infiltration targeted 3–6 cotyledons of each *N. benthamiana* plant (as described in [51]). Post-infiltration, the *N. benthamiana* plants were placed in a light incubator for 8 days at 24 °C in darkness, after which the flavonoid content in the leaves was measured using a detection kit (Solarbio, Beijing, China). Three biological replicates were prepared for each transient overexpression analysis, and the primer sequences are provided in Table S5.

### 4.8. Bioinformatics Analysis

ProtParam (https://web.expasy.org/protparam/, accessed on 1 June 2020) was utilized to analyze the fundamental properties of the protein sequences. Prot Scale (http://web.expasy.org/protscale/, accessed on 1 June 2020) was employed to predict the hydrophobicity and hydrophilicity of the amino acid sequences. The transmembrane structure of the proteins was analyzed using TMHMM Server v. 2.0 (http://www.cbs.dtu.dk/services/TMHMM/, accessed on 18 May 2020). SignalP 4.1 Server (http://www.cbs.dtu.dk/services/SignalP-4.1/, accessed on 5 June 2020) was used to predict signal peptides within the amino acid sequences. Finally, Plant-Ploc (http://www.csbio.sjtu.edu.cn/bioinf/plant/, accessed on 13 May 2020) was employed to predict the subcellular localization of the proteins.

### 4.9. Statistical Analysis

The data were analyzed using a one-way analysis of variance (ANOVA) and Duncan’s multiple range test (*p* < 0.05) using SPSS 22.0 Statistics (SPSS Inc., Chicago, IL, USA). The volcano plots, MA map, K-means cluster analysis and heatmap were created using software available online at https://cloud.metware.cn/#/tools/tool-list, accessed on 25 May 2020. OPLS-DA was carried out using online software (https://www.omicshare.com/tools/Home/Soft/getsoft, accessed on 25 May 2020). Correlation analysis was performed using BMKCloud (https://www.biocloud.net accessed on 28 May 2020). Graphs were drawn using OriginPro 2021 (OriginLab, Northampton, MA, USA).

## 5. Conclusions

In this study, transcriptomic and metabolomic analyses were conducted to investigate the mechanisms underlying fruit color in *Z. bungeanum.* A total of 137 flavonoids were identified. Among these, tricetin and (-)-epigallocatechin were the most significantly upregulated and downregulated flavonoids across all developmental stages, respectively, indicating their critical roles in the color change of *Z. bungeanum*. Additionally, nine DEGs, twelve MYB TFs, and three bHLH TFs were identified, suggesting they may work synergistically to regulate the structural genes in the flavonoid biosynthesis pathway of *Z. bungeanum*. The transient expression of key genes in flavonoid biosynthesis (*ZbANR*, *ZbFLS*, and *ZbCHI*) all led to an increased flavonoid content in *N. benthamiana*, with *ZbANR* overexpression resulting in a pronounced white phenotype. Together, this study not only provides new insights into the understanding of the synthesis and accumulation of flavonoids in *Z. bungeanum*, but also serves as a significant reference for breeding improved varieties of this species.

## Figures and Tables

**Table 1:** The 20 metabolites with the most significant differences in the a–b period.

Compounds	Class	CAS	VIP	Fold_Change	LogFC	Type
Tricetin	Flavonoid	520-31-0	4.227209912	1228.811111	10.26304745	up
Cyanidin 3-rutinoside(Keracyanin chloride)	Anthocyanins	18719-76-1	2.50252972	11.97053496	3.581415723	up
Laricitrin-O-acetylhexoside	Flavonoid	-	2.317105575	9.890858111	3.306095692	up
Cyanidin O-syringic acid	Anthocyanins	-	2.299356234	8.124485792	3.022276506	up
Cyanidin 3-O-galactoside	Anthocyanins	27661-36-5	2.247370438	7.430768054	2.893511337	up
Hesperetin	Dihydroflavone	520-33-2	1.9095353	4.306970769	2.106673532	up
Chrysoeriol 6-C-hexoside 8-C-hexoside-O-hexoside	Flavonoid	-	1.733682182	3.819869272	1.933523265	up
Jaceosidin	Anthocyanins	18085-97-7	1.814117738	3.736101702	1.901533728	up
Syringetin	Flavonols	4423-37-4	1.500606915	2.502910686	1.323606811	up
Apigeninidin chloride	Flavonoid	28590-40-1	1.515537169	2.501247549	1.322647849	up
Diosmetin	Flavonoid	520-34-3	1.284414289	2.243455135	1.165722333	up
Fustin	Dihydroflavonol	20725-03-5	1.321594673	0.483519473	−1.0483541	down
Di-O-methylquercetin	Flavonols	2068-02-2	1.338157017	0.466483907	−1.100100783	down
Luteolin	Flavonoid	491-70-3	1.321313536	0.458803225	−1.124052563	down
Rhoifolin	Flavonoid	17306-46-6	1.422257814	0.443161521	−1.174095475	down
3-O-Acetylpinobanksin	Dihydroflavonol	52117-69-8	1.408512593	0.425530777	−1.232664615	down
Taxifolin	Dihydroflavonol	480-18-2	1.802615427	0.273097387	−1.872512583	down
(+)-Gallocatechin	Flavanols	970-73-0	1.917437534	0.230976976	−2.114179047	down
Catechin	Flavanols	154-23-4	1.919967889	0.225461861	−2.149044687	down
(-)-Epigallocatechin	Flavanols	970-74-1	2.067616727	0.182804019	−2.451630303	down

CAS: Chemical Abstracts Service number; VIP: variable important in projection; Fold_Change: the fold change in metabolite expression during the a–b period; LogFC: log fold change.

## Data Availability

The original contributions presented in the study are included in the article/Supplementary Materials, further inquiries can be directed to the corresponding author.

## Supplementary Materials

The following supporting information can be downloaded at https://www.mdpi.com/article/10.3390/plants14040558/s1: Figure S1: KEGG pathway enrichment; Figure S2: GO classification of differentially expressed genes; Figure S3: DEG KEGG annotation; Figure S4: KEGG pathway enrichment of DEGs; Figure S5: The main metabolites (relative content) during the ripening of *Z. bungeanum*; Figure S6: qPCR validation of 10 DEGs of *Z. bungeanum*; Figure S7: Amplification of CDSs; Figure S8: Deduced amino acid sequences of the conserved regions of *ZbCHI*; Figure S9: Deduced amino acid sequences of the conserved regions of *ZbFLS*; Figure S10: Deduced amino acid sequences of the conserved regions of *ZbANR*; Figure S11: Hydrophobicity and hydrophilicity prediction protein, (a) ZbCHI, (b) ZbFLS, and (c) ZbANR; Figure S12: Analysis of protein transmembrane structure, (a) ZbCHI, (b) ZbFLS, and (c) ZbANR; Figure S13: Subcellular localization prediction, (a) ZbCHI, (b) ZbFLS, and (c) ZbANR; Table S1: Flavonoids detected in this experiment; Table S2: Transcriptome sequencing results; Table S3: Statistics of transcription splicing results; Table S4: Gene function annotation; Table S5: Primers in this study.

## References

[1] Hernandez I., Alegre L., Van B., Munne-Bosch S. (2009). How relevant are flavonoids as antioxidants in plants?. Trends Plant Sci..

[2] Wang L., Chen M., Lam P., Dini-Andreote F., Dai L., Wei Z. (2022). Multifaceted roles of flavonoids mediating plant-microbe interactions. Microbiome.

[3] Pucker B., Reiher F., Schilbert H. (2020). Automatic identification of players in the flavonoid biosynthesis with application on the biomedicinal plant *Croton tiglium*. Plants.

[4] Dretcanu G., Stirbu I., Leoplold N., Cruceriu D., Danciu C., Stanila A., Farcas A., Borda I., Iuhas C., Diaconeasa Z. (2022). Chemical structure, sources and role of bioactive flavonoids in cancer prevention: A review. Plants.

[5] Rahaman M., Rakib A., Mitra S., Tareq A., Emran T., Shahid-Ud-Daula A., Amin M., Simal-Gandara J. (2021). The genus Curcuma and inflammation: Overview of the pharmacological perspectives. Plants.

[6] Du H., Ke J., Sun X., Tan L., Yu Q., Wei C., Ryan P., Wang A., Li H. (2024). FtMYB163 gene encodes SG7 R2R3-MYB transcription factor from tartary buckwheat (*Fagopyrum tataricum* Gaertn.) to promote flavonol accumulation in transgenic *Arabidopsis thaliana*. Plants.

[7] Chong Y., Kim B., Park Y., Yang Y., Lee S., Lee Y., Ahn J. (2023). Production of four flavonoid C-Glucosides in *Escherichia coli*. J. Agric. Food Chem..

[8] Wang F., Lin K., Shen Q., Liu D., Xiao G., Ma L. (2024). Metabolomic analysis reveals the effect of ultrasonic-microwave pretreatment on flavonoids in tribute Citrus powder. Agric. Food Chem..

[9] Gu Z., Men S., Zhu J., Hao Q., Tong N., Liu Z., Zhang H., Shu Q., Wang L. (2019). Chalcone synthase is ubiquitinated and degraded via interactions with a ring-h2 protein in petals of paeonia ‘he xie’. J. Exp. Bot..

[10] Lv Y., Li D., Wu L., Zhu Y., Ye Y., Zheng X., Lu J., Liang Y., Li Q., Ye J. (2022). Sugar signal mediates flavonoid biosynthesis in tea leaves. Hortic. Res..

[11] Cheng H., Li L., Cheng S., Cao F., Wang Y., Yuan H. (2011). Molecular cloning and function assay of a chalcone isomerase gene (gbchi) from *Ginkgo biloba*. Plant Cell Rep..

[12] Guo J., Zhou W., Lu Z., Li H., Li H., Gao F. (2015). Isolation and functional analysis of chalcone isomerase gene from purple-fleshed sweet potato. Plant Mol. Biol. Rep..

[13] Wu Y., Zhu M., Jiang Y., Zhao D., Tao J. (2018). Molecular characterization of chalcone isomerase (chi) regulating flower color in herbaceous peony (*Paeonia lactiflora* pall.). J. Integr. Agric..

[14] Liu T., Liu H., Xian W., Liu Z., Yuan Y., Fan J., Xiang S., Yang X., Liu Y., Liu S. (2024). Duplication and sub-functionalization of flavonoid biosynthesis genes plays important role in Leguminosae root nodule symbiosis evolution. J. Integr. Plant Biol..

[15] Zhou L., Wang Y., Ren L., Shi Q., Zheng B., Miao K., Guo X. (2014). Overexpression of ps-chi1, a homologue of the chalcone isomerase gene from tree peony (*Paeonia suffruticosa*), reduces the intensity of flower pigmentation in transgenic tobacco. Plant Cell Tiss. Org..

[16] Zhou X., Fan Z., Chen Y., Zhu Y., Li J., Yin H. (2013). Functional analyses of a flavonol synthase-like gene from *Camellia nitidissima* reveal its roles in flavonoid metabolism during floral pigmentation. J. Biosci..

[17] Luo P., Ning G., Wang Z., Shen Y., Jin H., Li P., Huang S., Zhao J., Bao M. (2016). Disequilibrium of flavonol synthase and dihydroflavonol-4-reductase expression associated tightly to white vs. Red color flower formation in plants. Front. Plant Sci..

[18] Xia X., Du H., Hu X., Wu J., Yang F., Li C., Huang S., Wang Q., Liang C., Wang X. (2024). Genomic insights into adaptive evolution of the species-rich cosmopolitan plant genus *Rhododendron*. Cell Rep..

[19] Luan A., Zhang W., Yang M., Zhong Z., Wu J., He Y., He J. (2023). Unveiling the molecular mechanism involving anthocyanins in pineapple peel discoloration during fruit maturation. Food Chem..

[20] Huang X., Chu G., Wang J., Luo H., Yang Z., Sun L., Rong W., Wang M. (2023). Integrated metabolomic and transcriptomic analysis of specialized metabolites and isoflavonoid biosynthesis in *Sophora alopecuroides* L. under different degrees of drought stress. Ind. Crop. Prod..

[21] Chen Y., Li W., Jia K., Liao K., Liu L., Fan G., Zhang S., Wang Y. (2023). Metabolomic and transcriptomice analyses of flavonoid biosynthesis in apricot fruits. Front. Plant Sci..

[22] Song Y., Zhang G., Chen N., Zhang J., He C. (2023). Metabolomic and transcriptomic analyses provide insights into the flavonoid biosynthesis in sea buckthorn (*Hippophae rhamnoides* L.). LWT-Food Sci. Technol..

[23] Liu Y., Lv J., Liu Z., Wang J., Yang B., Chen W., Ou L., Dai X., Zhang Z., Zou X. (2020). Integrative analysis of metabolome and transcriptome reveals the mechanism of color formation in pepper fruit (*Capsicum annuum* L.). Food Chem..

[24] Shi J., Fei X., Hu Y., Liu Y., Wei A. (2019). Identification of key genes in the synthesis pathway of volatile terpenoids in fruit of *Zanthoxylum bungeanum* Maxim. Forests.

[25] Tian L., Shi J., Yang L., Wei A. (2022). Molecular cloning and functional analysis of *DXS* and *FPS* Genes from *Zanthoxylum bungeanum* Maxim. Foods.

[26] Ma Y., Li X., Hou L., Wei A. (2019). Extraction solvent affects the antioxidant, antimicrobial, cholinesterase and HepG2 human hepatocellular carcinoma cell inhibitory activities of *Zanthoxylum bungeanum* pericarps and the major chemical components. Ind. Crop. Prod..

[27] Lu D., Lo V. (2015). Scent and synaesthesia: The medical use of spice bags in early China. J. Ethnopharmacol..

[28] Sun J., Sun B., Ren F., Chen H., Zhang N., Zhang Y. (2020). Characterization of key odorants in hanyuan and hancheng fried pepper (*Zanthoxylum bungeanum*) oil. J. Agric. Food Chem..

[29] Yu L., Wu W., Pan Y., Wang W., Sun L., Liu Y., Wang D., Li D. (2020). Quality evaluation of different varieties of *Zanthoxylum bungeanum* Maxim. Peels based on phenolic profiles, bioactivity, and HPLC fingerprint. J. Food Sci..

[30] Sun L., Yu D., Wu Z., Wang C., Yu L., Wei A., Wang D. (2019). Comparative transcriptome analysis and expression of genes reveal the biosynthesis and accumulation patterns of key flavonoids in different varieties of *Zanthoxylum bungeanum* leaves. J. Agric. Food Chem..

[31] Chen X., Wang W., Wang C., Liu Z., Sun Q., Wang D. (2019). Quality evaluation and chemometric discrimination of *Zanthoxylum bungeanum* maxim leaves based on flavonoids profiles, bioactivity and hplc-fingerprint in a common garden experiment. Ind. Crop. Prod..

[32] Varet H., Brillet Gueguen L., Coppee J., Dillies M. (2016). Sartools: A deseq2-and edger-based r pipeline for comprehensive differential analysis of rna-seq data. PLoS ONE.

[33] Zhang X., Ivanova A., Vandepoele K., Radomiljac J., Van de Velde J., Berkowitz O., Willems P., Xu Y., Ng S., Van Aken O. (2017). The transcription factor MYB29 is a regulator of *ALTERNATIVE OXIDASE1a*. Plant Physiol..

[34] Jiang L., Yue M., Liu Y., Zhang N., Lin Y., Zhang Y., Wang Y., Li M., Luo Y., Zhang Y. (2023). A novel R2R3-MYB transcription factor FaMYB5 positively regulates anthocyanin and proanthocyanidin biosynthesis in cultivated strawberries (*Fragaria x ananassa*). Plant Biotechnol. J..

[35] Chang P., Hsieh M., Hsieh Y., Chen P., Yang J., Lo F., Yang S., Lu K. (2017). Tricetin inhibits human osteosarcoma cells metastasis by transcriptionally repressing MMP-9 via p38 and Akt pathways. Environ. Toxicol..

[36] Hsu Y., Uen Y., Chen Y., Liang H., Kuo P. (2009). Tricetin, a dietary flavonoid, inhibits proliferation of human breast adenocarcinoma mcf-7 cells by blocking cell cycle progression and inducing apoptosis. J. Agric. Food Chem..

[37] Wu J., Wang X., He Y., Li J., Ma K., Zhang Y., Li H., Yin C., Zhang Y. (2022). Stability evaluation of gardenia yellow pigment in presence of different phenolic compounds. Food Chem..

[38] Tian J., Han Z., Zhang J., Hu Y., Song T., Yao Y. (2015). The balance of expression of dihydroflavonol 4-reductase and flavonol synthase regulates flavonoid biosynthesis and red foliage coloration in Crabapples. Sci. Rep..

[39] Wang Y., Zhou L., Song A., Wang Y., Geng Z., Zhao K., Jiang J., Chen S., Chen F. (2023). Comparative transcriptome analysis and flavonoid profiling of floral mutants reveals CmMYB11 regulating flavonoid biosynthesis in chrysanthemum. Plant Sci..

[40] Xu P., Li M., Ma C., Li X., Bai P., Lin A., Wang C., Zhang L., Kuang H., Lian H. (2024). Loss-of-function mutation in anthocyanidin reductase activates the anthocyanin synthesis pathway in strawberry. Mol. Hortic..

[41] Liu C., Wang X., Shulaev V., Dixon R.A. (2016). A role for leucoanthocyanidin reductase in the extension of proanthocyanidins. Nat. Plants.

[42] Zhang Q., Wang L., Liu Z., Zhao Z., Zhao J., Wang Z., Zhou G., Liu P., Liu M. (2020). Transcriptome and metabolome profiling unveil the mechanisms of *Ziziphus jujuba* Mill. Peel coloration. Food Chem..

[43] Wan L., Lei Y., Yan L., Liu Y., Pandey M., Wan X., Varshney R., Fang J., Liao B. (2020). Transcriptome and metabolome reveal redirection of flavonoids in a white testa peanut mutant. BMC Plant Biol..

[44] Tang K., Karamat U., Li G., Guo J., Jiang S., Fu M., Yang X. (2024). Integrated metabolome and transcriptome analyses reveal the role of *BoGSTF12* in anthocyanin accumulation in Chinese kale (*Brassica oleracea var. alboglabra*). BMC Plant Biol..

[45] Ma C., Meng L., Wang R., Fan Y., Wang R. (2022). Dynamics of anthocyanin profiles of the fruits of four blueberry (*Vaccinium* sp.) cultivars during different growth stages. Int. J. Food Prop..

[46] Liu X., Yang W., Mu B., Li S., Li Y., Zhou X., Zhang C., Fan Y., Chen R. (2018). Engineering of ‘purple embryo maize’ with a multigene expression system derived from a bidirectional promoter and self-cleaving 2a peptides. Plant Biotechnol. J..

[47] Nemesio-Gorriz M., Blair P., Dalman K., Hammerbacher A., Arnerup J., Stenlid J., Mukhtar S.M., Elfstrand M. (2017). Identification of norway spruce myb-bhlh-wdr transcription factor complex members linked to regulation of the flavonoid pathway. Front. Plant Sci..

[48] Qi T., Huang H., Song S., Xie D. (2015). Regulation of jasmonate-mediated stamen development and seed production by a bhlh-myb complex in Arabidopsis. Plant Cell.

[49] Shi J., Deng L., Gunina A., Alharbi S., Wang K., Li J., Liu Y., Shangguan Z., Kuzyakov Y. (2023). Carbon stabilization pathways in soil aggregates during long-term forest succession: Implications from δ^13^C signatures. Soil Biol. Biochem..

[50] Shi J., Song M., Yang L., Zhao F., Wu J., Li J., Yu Z., Li A., Shangguan Z., Deng L. (2023). Recalcitrant organic carbon plays a key role in soil carbon sequestration along a long-term vegetation succession on the Loess Plateau. Catena.

[51] Salvatierra A., Pimentel P., Alejandra Moya-Leon M., Herrera R. (2013). Increased accumulation of anthocyanins in *Fragaria chiloensis* fruits by transient suppression of fcmyb1 gene. Phytochemistry.

