# Supplementary material for: Transcriptome and Metabolome Profiling Provide Insights into Flavonoid Biosynthesis and the Mechanism of Color Formation in Zanthoxylum bungeanum Maxim
Source: Plants (Basel). 2025 Feb 12;14(4):558. doi: 10.3390/plants14040558 (PMC11858979; doi:10.3390/plants14040558)
Supplement: Supplementary file 1 [file plants-14-00558-s001.zip › plants-3307640-supplementary.pdf]

Supplemental Figures and Tables

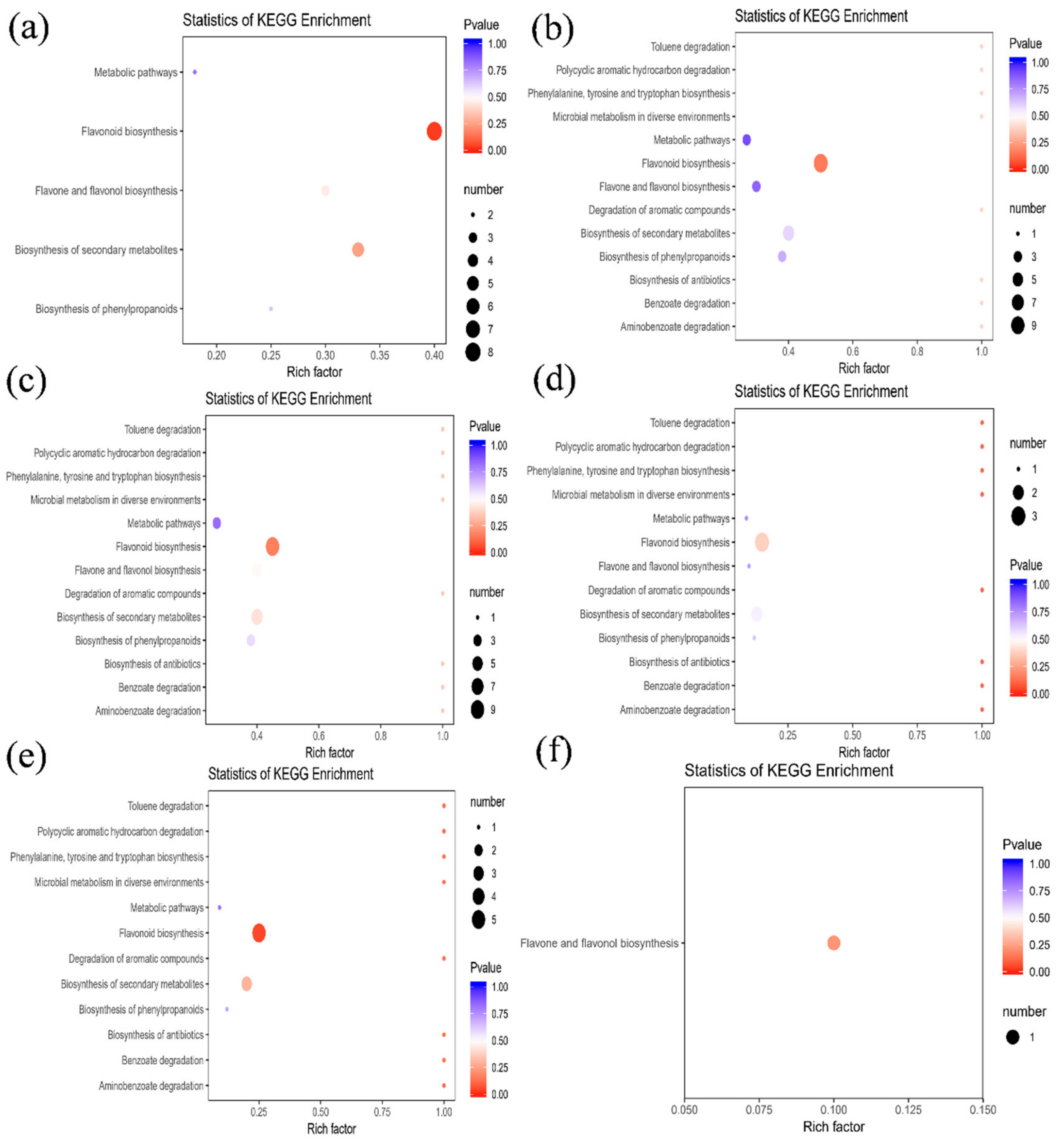

Figure S1. KEGG pathway enrichment. (a) a vs b; (b) a vs c; (c) a vs d; (d) b vs c; (e) b vs d; (f) c vs d. The abscissa represents the rich factor corresponding to each pathway, the ordinate represents the name of the pathway, the color of the point is pvalue, and the redder the enrichment, the more significant. The point size represents the number of differential metabolites enriched.

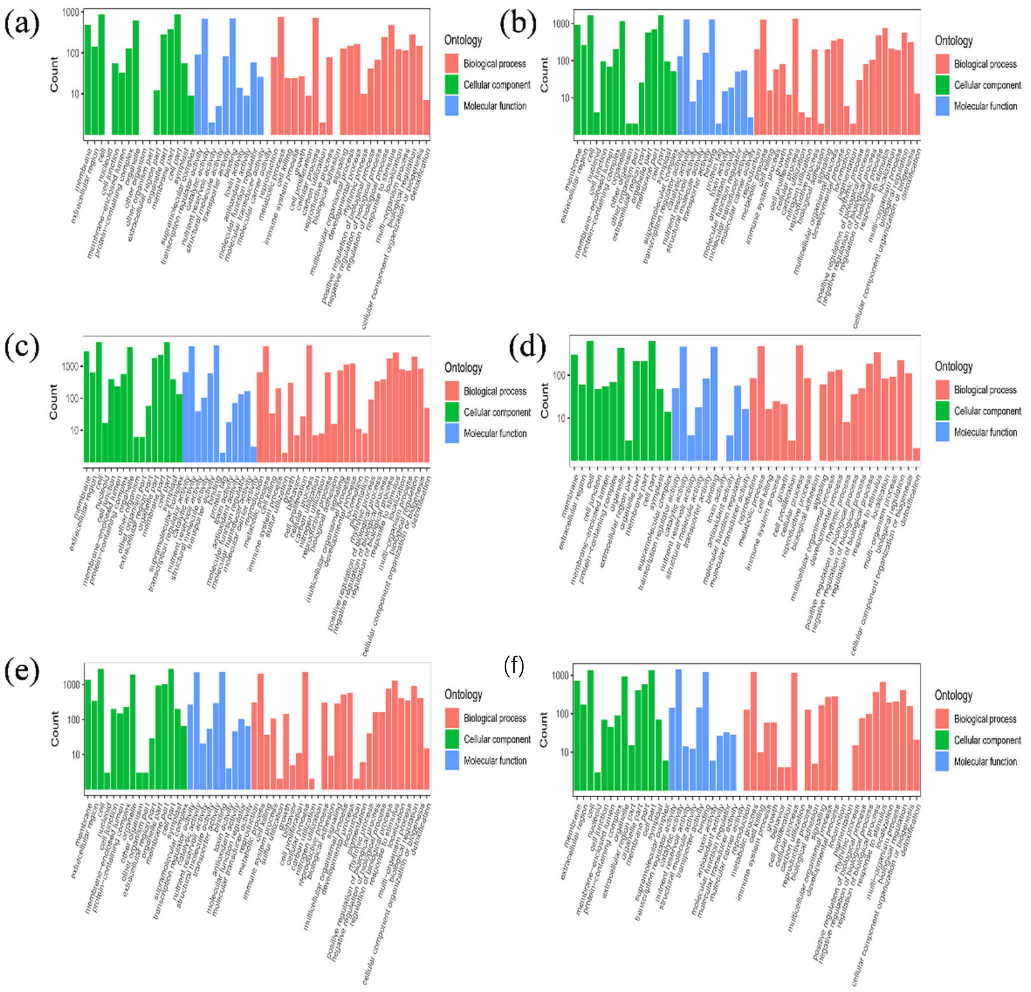

Figure S2. GO classification of differentially expressed genes. (a) A vs B; (b) A vs C; (c) A vs D; (d) B vs C; (e) B vs D; (f) C vs D. The ordinate represents the second-level GO entry, and the abscissa represents the ratio of the number of different genes to the total number of different genes.

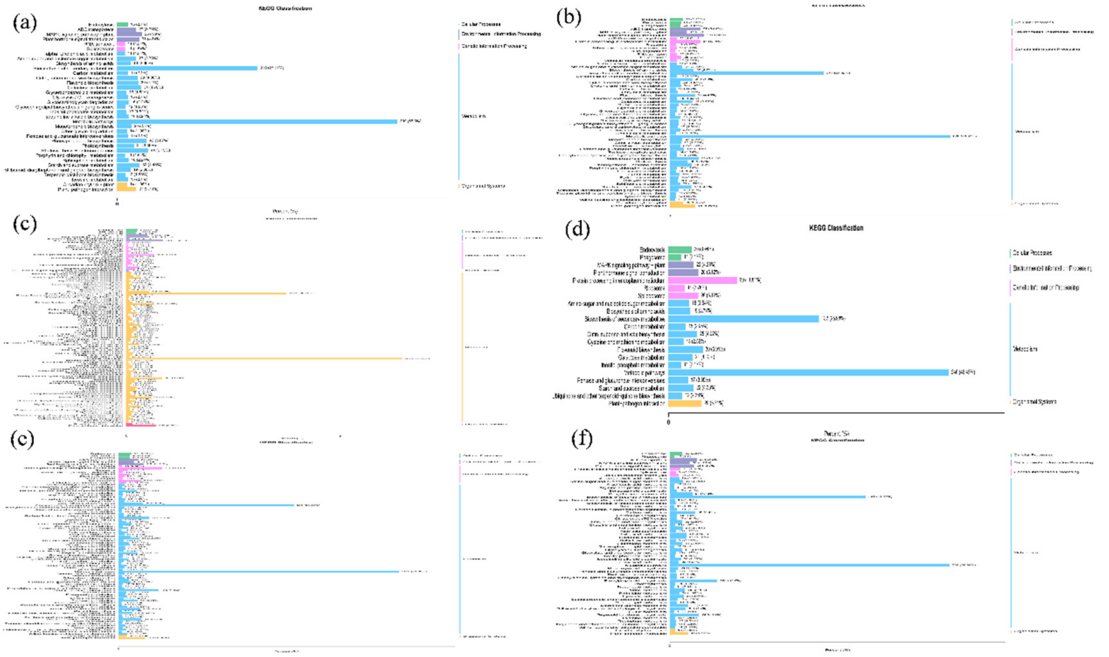

Figure S3. DEGs KEGG annotation. (a) A vs B; (b) A vs C; (c) A vs D; (d) B vs C; (e) B vs D; (f) C vs D. The x-coordinate represents the ratio of genes annotated to the total number of genes annotated, and the y-coordinate represents the name of the KEGG pathway. The label on the right of the figure represents the category to which the KEGG pathway belongs.

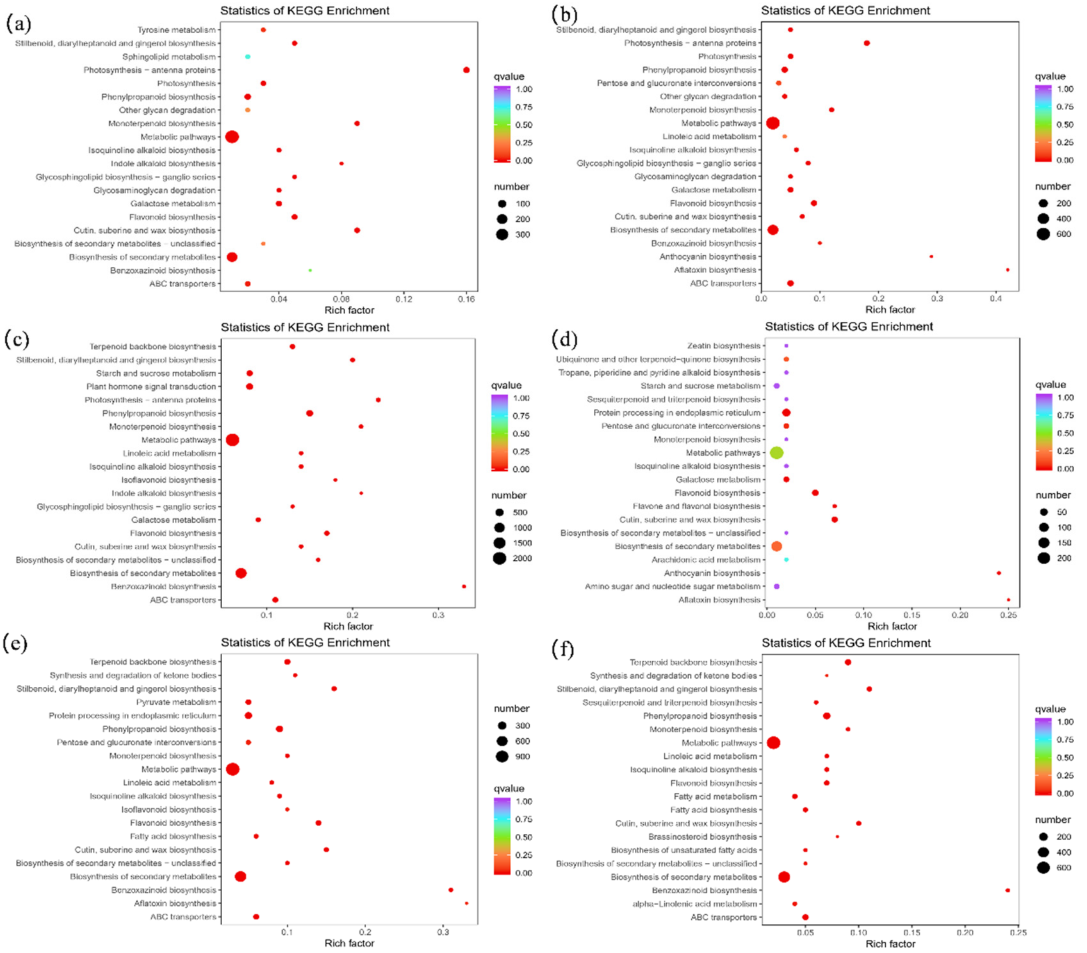

Figure S4. KEGG pathway enrichment of DEGs. (a) A vs B; (b) A vs C; (c) A vs D; (d) B vs C; (e) B vs D; (f) C vs D. The ordinate represents the KEGG path. The abscissa is rich factor. The greater the rich factor is, the greater the enrichment degree will be. The larger the point, the greater the number of DEGs enriched in the pathway. The redder the dot color, the more significant the enrichment.

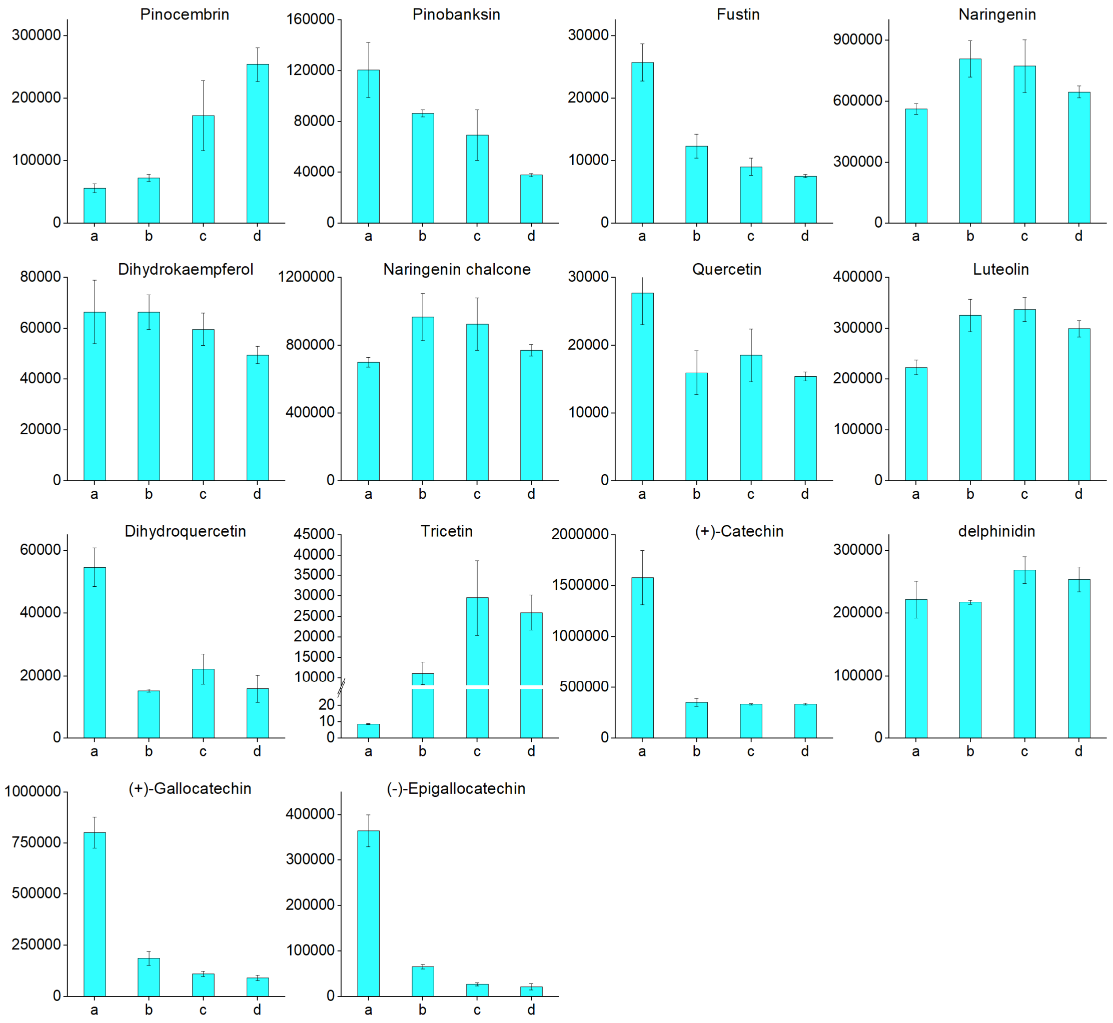

Figure S5. The mian metabolites (relative content) during ripening of *Z. bungeanum*. Each bar represents the mean ± SD of three biological replicates.

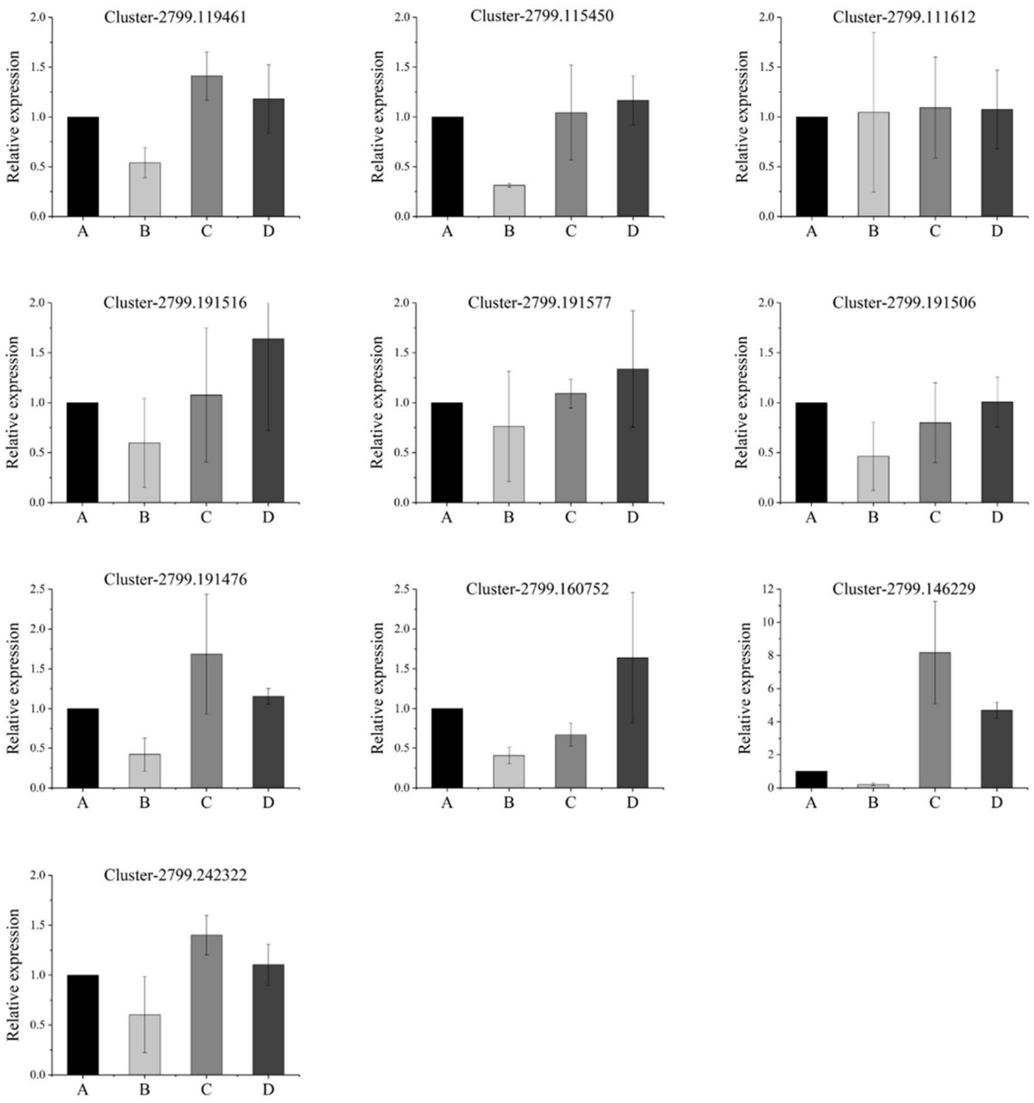

Figure S6. qPCR validation of 10 DEGs of *Z. bungeanum*. The bar chart showed Relative expression results, each bar represents the mean ± SD of three biological replicates.

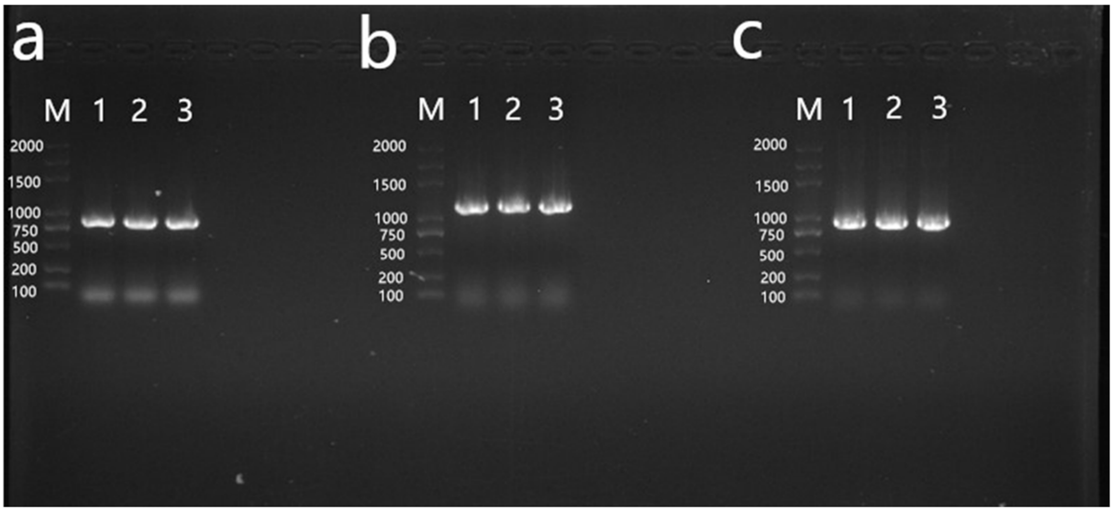

Figure S7. Amplification of CDSs. (a) *ZbANR*; (b) *ZbCHI*; (c) *ZbFLS*. M: DNA marker (DL2000), 1-3: PCR products.

```
Citrus_sinensis          MGNNW.FFMDFNGGDPYILPTEFFLARSLGDHLFSQSS..   37
Citrus_clementina        MGNNW.FFMDFNGGDPYILPTEFFLARSLGDHLFSQSS..   37
Trema_orientale          MRNNWLWFMDLDGGSPYIFPVEPIAMNNFGCHQFSQISSF   40
Macleaya_cordata         MPNNWLFFLDLDGESPYNFPTEPVVS..............   26
Zanthoxylum_bungeanum    MG.....FMDFDGGDPYILPTQFFLSRSLGGHLFSQSS..   33
Consensus                m      f d  g  py  p

Citrus_sinensis          .........YPYVPGSLALQGAFNCISKFTGAFLFWFTSG   68
Citrus_clementina        .........YPYVPGSLALQGAFNCISKFTGAFLFWFTSG   68
Trema_orientale          MDNSFYQYRHLSIPGSLAIEGAFNHIAKLAGALLLVFSSG   80
Macleaya_cordata         ..............GSLAVQEAFNCISKLAGALTFWFASG   52
Zanthoxylum_bungeanum    .........YLYGPGSLALQDAFNCISKFTGAFLFWFTSG   64
Consensus                              gsla   afn i k  ga    f sg

Citrus_sinensis          SSSNLSRRIAGNQHGSKIGSYKSSAQVKQITSTGHNLTGF   108
Citrus_clementina        SSSNLSRRIAGNQHGSKIGSYKSSAQVKQITSTGHNLTGF   108
Trema_orientale          SSLNLTREITGLPHGPKPRSCRSFSQVKHIASSRQNFRKL   120
Macleaya_cordata         SNSILHHKLSGNPHGSNPGSSESRSLVKRITSCEHNLAGL    92
Zanthoxylum_bungeanum    SSSNLSRKIAGNQHGSKPGSYKTPAQVKQITATGHNLAGF   104
Consensus                s   l     g  hg    s      vk i     n

Citrus_sinensis          HLSSNSESEYACPVVFNKVSSSAIRRLFREGGKLHLSSVL   148
Citrus_clementina        HLSSNSESEYACPVVFNKVSSSAIRRLFREGGKLHLSSVL   148
Trema_orientale          RFGFRSQGESGMPVIFGKISSFVSKLLL..AERLQSHSFL   158
Macleaya_cordata         QFGSRSRAEPAAHVLFGKFVRFTMRHILKEVEQLRSFSVL   132
Zanthoxylum_bungeanum    HFGSNSESEYASPVVFCKVSNSAIKCLFREGGSLQLSSVL   144
Consensus                     s  e    v f k               l   s l

Citrus_sinensis          SLAAAVIPPLDNVSSKVLAVPLDNAEIQMHESMGRSPCEV   188
Citrus_clementina        SLAAAVIPPLDNVSSKVLAVPLDNAEIQMHESMGRSPCEV   188
Trema_orientale          SLAAALVPPLDNISSKVLAVPLENSEVMV.....QRSCEV   193
Macleaya_cordata         SLAAALVPPFDNLSLKVHAVPLENADIQINGLMDQRPCMA   172
Zanthoxylum_bungeanum    SLAAAVIPPFENVSSKALAVPLGNAEIQMHESMGRSPCEV   184
Consensus                slaaa  pp  n s k  avpl n             c

Citrus_sinensis          ESHRCGALSFPCLSRTRHAVEPRTGIEFPMILDNVIDGEN   228
Citrus_clementina        ESHRCGALSFPCLSRTRHAVEPRTGIEFPMILDNVIDGEN   228
Trema_orientale          EHQGRAGLSFPCLNWRRHAVEPRTGIEFPFILHNILAGEN   233
Macleaya_cordata         EHRGSSNLSFHEIDWTRHAVEPRTGIEFPTVLDNNLAEGN   212
Zanthoxylum_bungeanum    ESHRCGALSFPCLSGTRPAVEPRTGIEFPMILDNTIDGEN   224
Consensus                e      lsf      r aveprtgiefp  l n     n

Citrus_sinensis          YSSSTSEVLVGTGSRTIKIVKIKSLKVYAFAFYVHPRSVC   268
Citrus_clementina        YSSSTSEVLVGTGSRTIKIVKIKSLKVYAFAFYVHPRSVC   268
Trema_orientale          NSNLCSEVLVGTGSRTMTIIRLKSLKVYAYGFYVHPSSVC   273
Macleaya_cordata         NSSFTSEVLVGTGFRSMRVIKFRSVKVYAFGLYVHPDSLC   252
Zanthoxylum_bungeanum    YSSSTSEVLVGTGSRTMKIVKIKSLKVYAFAFYVHPRSVC   264
Consensus                 s   sevlvgtg r        s kvya   yvhp s c

Citrus_sinensis          EKLGPKYASISVAELNKCGGFYEDLLREDIDMTVRLVINC   308
Citrus_clementina        EKLGPKYASISVAELNKCGGFYEDLLREDIDMTVRLVINC   308
Trema_orientale          EKLGKKYASFSVDELNTRQDFYEDLLREDINMTVRLVVSC   313
Macleaya_cordata         EKLGWKYASVPTGELNNCPDFLQDLLREDIDMTVRLVVNC   292
Zanthoxylum_bungeanum    EKLGSKYASIPVGELNKCCGFYEDLLREDIDMTVRLVINC   304
Consensus                eklg kyas    eln    f  dllredi mtvrlv  c

Citrus_sinensis          NGMKVNAVKDVFEKSLRARLEKVN.HLAYGITLVF.....   342
Citrus_clementina        NGMKVNAVKDVFEKSLRARLEKANPNTDYHCLESFGSYFS   348
Trema_orientale          NGLKINSVKDAFEKSLRARLVKTNPDTDYHCIGSFGSCFT   353
Macleaya_cordata         NGLKINTVRDAFEKSLRARLLKMNPNANCHCLRTFGSYFT   332
Zanthoxylum_bungeanum    NGMKVNAVKDAFEKSLRARLEKANPNTDYHCLKTFGSYFT   344
Consensus                ng k n v d fekslrarl k n          f

Citrus_sinensis          LQISLTHGTFLAYIRCIQTLIITAW...KVLVITSAKIFH   379
Citrus_clementina        KDIPLPLGTVINFRRTAAGCLITEIGGNQIGAVHSKCLCR   388
Trema_orientale          QDIPLPAGTTIHFRRTADGCLITEIGGNQIGAVQSKCLCR   393
Macleaya_cordata         HNIPLPAGTTIDFRRTVDGCLITRIGGKQIGAVHSKCLCR   372
Zanthoxylum_bungeanum    KDIPLPRGTIINFRRTSAGCLITEIGGNQIGAVDSRCLCR   384
Consensus                  i l  gt     r      it           s

Citrus_sinensis          YLWEL.........................             384
Citrus_clementina        AFFDMYIGTAPVSEQTKEEIGKNVASMIKT             418
Trema_orientale          AFFSMYIGDVPVSEQTKVEIGRNVANLIRR             423
Macleaya_cordata         AFFDMYIGDVPVSVQAKEEICRNVGSILRR             402
Zanthoxylum_bungeanum    AFFDMYLGTTPVSEQTKEEIGKNVASLVTR             414
Consensus
```

Figure S8. Deduced amino acid sequences of the conserved regions of *ZbCHI*. Homology level was highlighted by shading in color: black for 100%, pink for ≥ 75% and blue for ≥ 50% identity.

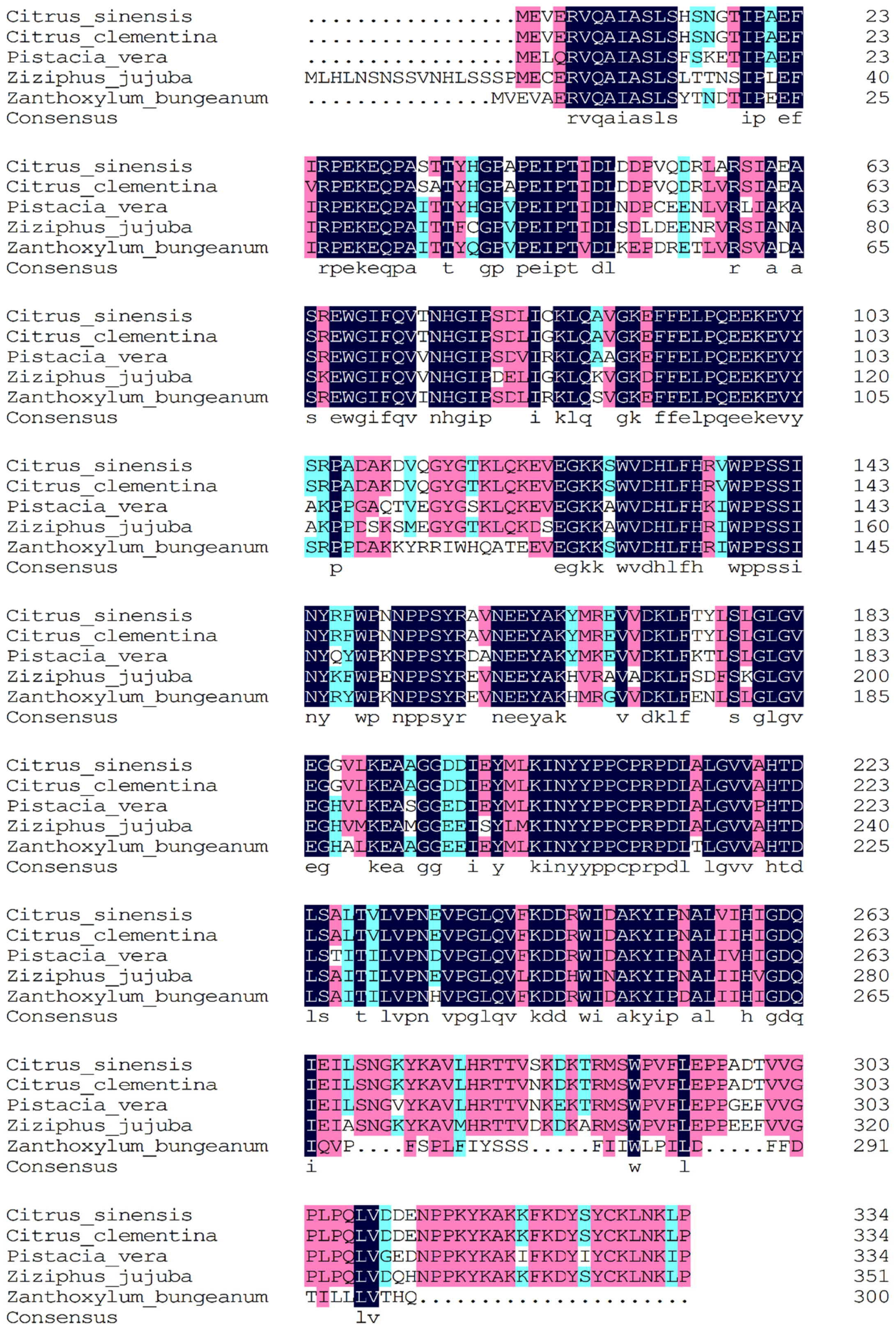

Figure S9. Deduced amino acid sequences of the conserved regions of *ZbFLS*. Homology level was highlighted by shading in color: black for 100%, pink for ≥ 75% and blue for ≥ 50% identity.

```
Citrus_clementina      MASQSTQKKTACVVGGTGFVASLLIKLLLHNGYAVHATVR   40
Pistacia_vera          MSAQLTEKKTACVVGGTGYVASLLIKLLLQKGYSVNTTVR   40
Citrus_sinensis        MASQSTQKKTACVVGGTGFVASLLIKLLLHNGYAVHATVR   40
Quercus_suber          MATQNLGKKTACVVGGTGFVASLLVKLLLQKGYAVNTTVR   40
Zanthoxylum_bungeanum  MASQFTQKKTACVVGGTGFVASLLIKLLLQKGYAVNTTVR   40
Consensus              m  q   kktacvvggtg vasll klll  gy v  tvr

Citrus_clementina      DPENQKKISPLIALQELGELKIFRADLTDEASFDAPISRS   80
Pistacia_vera          HPDNQKKIAHLISLQELGELKIFGADLTDEGSFDVPIAGS   80
Citrus_sinensis        DPENQKKISPLIALQELGELKIFRADLTDEASFDSPISGS   80
Quercus_suber          DPENKRKISHLIELQGLGDLKIFGADLTDEVSFDAPIAGC   80
Zanthoxylum_bungeanum  DPENQKMISHLTALQELGELKIFGANLTDEGSFDAPVSGS   80
Consensus               p n   i  l  lq lg lkif a ltde sfd p

Citrus_clementina      DIVFHVATPVNFSSDDPETDMIKPAIQGVVNVLKACTKTK  120
Pistacia_vera          DIVFHVATPVNFASEDPENDMIKPAIQGVVNVLKACAKSK  120
Citrus_sinensis        DIVFHVATPVNFSSDDPETDMIMPAIQGVVNVLKACTKTK  120
Quercus_suber          DLVFHIATPVNFASQDPENDMIKPAIQGVHNVLKACVRAK  120
Zanthoxylum_bungeanum  EIVFHVATPVNFASENPEDDMIKPAIKGVVNVLKACTKAK  120
Consensus                vfh atpvnf s  pe dmi pai gv nvlkac   k

Citrus_clementina      TVKRVILTSSAAAVSINAQNVTGLVMDEKNWTDVESLSSE  160
Pistacia_vera          TVKRVVLTSSTTAVTINGQNGTGLVMDEKNWTDVEFLSSA  160
Citrus_sinensis        TVARVILTSSAAAVSINAQNVTGLVMGEKNWTDVEFLSSE  160
Quercus_suber          TVKRVILTSSAAAVTINKLNGTGLVMDESKWTDVEFLSTE  160
Zanthoxylum_bungeanum  TVKRVILTSSAAAVSINAHNETGLVMDENNWTDVEFLSSE  160
Consensus              tv rv ltss  av in  n tglvm e  wtdve ls

Citrus_clementina      KPPTWGYAASKTLAERAACKFAQENNIDLITVIPSLMSGP  200
Pistacia_vera          KPPTWGYPASKTLAEKHAWKFAEENNIDLITIIPSLMAGP  200
Citrus_sinensis        KPPTWGYAASKTLAERAAWKFAQENNIDLITVIPSLMSGP  200
Quercus_suber          KPPTWGYPASKTLAEKAAWKFAEENNIDLITVIPTLMAGA  200
Zanthoxylum_bungeanum  KPPTWGYPASKALAEKAAWKFAQENNIDLITVIPSLMTGP  200
Consensus              kpptwgy ask lae  a kfa ennidlit ip lm g

Citrus_clementina      SLTPDIPSSVALAATLITGNDFLLNGLKGMQMLSGSISIA  240
Pistacia_vera          SLTPDIPSSTGLAMCLITGNEFLTNALKGMQMLSGSISIA  240
Citrus_sinensis        SLTPEIPSSVALAATLITGNEFLLNDLKGMQMLSGSISIA  240
Quercus_suber          SLTPDVPSSVGLAMSLITGTEFLINALKGMQMLSGSISIT  240
Zanthoxylum_bungeanum  SMTPDIPSSVALGASLITGNKFLINGLKGMQMLSGSISIS  240
Consensus              s tp  pss  l   litg  fl n lkgmqmlsgsisi

Citrus_clementina      HVEDVCRAHIFLAEKESASGRYICCAVNTSVPELAKFLNK  280
Pistacia_vera          HVEDVCRAHIFLAEKESASGRYICCAENTSVPELAKFLHQ  280
Citrus_sinensis        HVEDVCRAHIFLAEKESASGRYICCAVNTSVPELAKFLNK  280
Quercus_suber          HVEDVCRAHIFLAEKESASGRYICCGINSSVPELAKFLNK  280
Zanthoxylum_bungeanum  HVEDVCRAHIFLAEKESASGRYICCAVNTSVPELAKFLNK  280
Consensus              hvedvcrahiflaekesasgryicc  n svpelakfl

Citrus_clementina      RFPEYKVPTDFGDFPSEAKLILSSEKLISEGFCFKYGFED  320
Pistacia_vera          RYPQYKVPTDFGDFPSKAKLVISSEKLISEGFSFKFGIEE  320
Citrus_sinensis        RFPEYKVPTE..............................  290
Quercus_suber          RYPQYNVPTDFGDFPSKAKLTLSSDKLIKEGFSFKYGIED  320
Zanthoxylum_bungeanum  RYPEYNVPT...............................  289
Consensus              r p y vpt

Citrus_clementina      IYDQTVEYLKTKGMLK                          336
Pistacia_vera          IYDQTLEYFKSKGVLN                          336
Citrus_sinensis        ................                          290
Quercus_suber          IYDQTLEYFKAKGLLQ                          336
Zanthoxylum_bungeanum  ................                          289
Consensus
```

Figure S10. Deduced amino acid sequences of the conserved regions of *ZbANR*. Homology level was highlighted by shading in color: black for 100%, pink for ≥ 75% and blue for ≥ 50% identity.

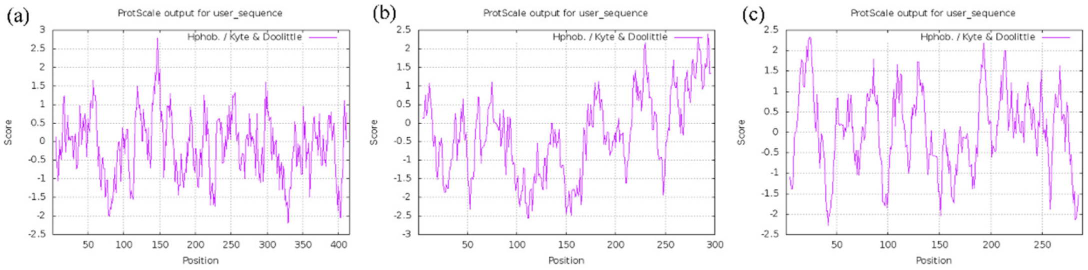

Figure S11. Hydrophobicity and hydrophilicity prediction protein. (a) ZbCHI; (b) ZbFLS; (c) ZbANR.

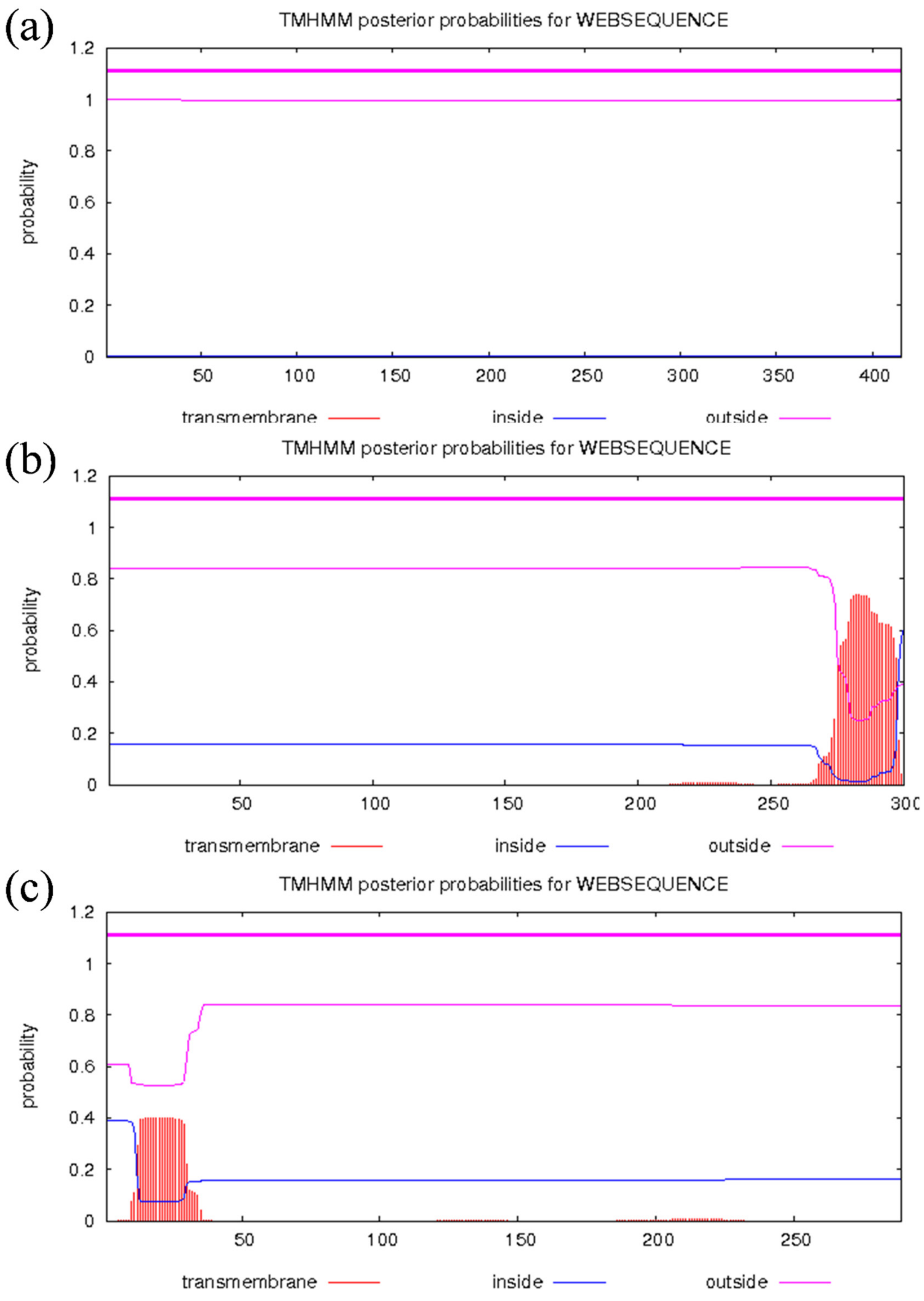

Figure S12. Analysis of protein transmembrane structure. (a) ZbCHI; (b) ZbFLS; (c) ZbANR.

(a) ----------- Plant-PLoc Computation Result -----------

Accession number	Predicted location(s)	Prediction approach
AZbCHI	**Chloroplast**	By fusing PseAA composition

(b) ----------- Plant-PLoc Computation Result -----------

Accession number	Predicted location(s)	Prediction approach
AZbFLS	**Cytoplasm**	By fusing PseAA composition

(c) ----------- Plant-PLoc Computation Result -----------

Accession number	Predicted location(s)	Prediction approach
AZbANR	**Plasma membrane**	By fusing PseAA composition

Figure S13. Subcellular localization prediction (a) ZbCHI; (b) ZbFLS; (c) ZbANR.

Table S1. Flavonoids detected in this experiment.

Index	Q1 (Da)	Rt (min)	Molecular Weight (Da)	Ionization model	Formula	Compounds	Class I	Class II
GQ512001	771.13	3.18	772.17	[M-H]-	$C_{33}H_{40}O_{21}$	Quercetin-O-rutinoside-hexose	Flavonoids	Flavonols
GQ512002	623.1	4.4	624.14	[M-H]-	$C_{28}H_{32}O_{16}$	Isorhamnetin-3-O-rutinoside	Flavonoids	Flavonols
GQ512005	595.16	4.32	594.132	[M+H]+	$C_{27}H_{30}O_{15}$	Kaempferol-3-O-glucoside-7-O-rhamnoside	Flavonoids	Flavonols
GQ512006	611.16	4.09	610.126	[M+H]+	$C_{27}H_{30}O_{16}$	Quercetin-3-O-glucoside-7-O-rhamnoside	Flavonoids	Flavonols
Li512111	519.11	4.95	520.1	[M-H]-	$C_{24}H_{24}O_{13}$	Isorhamnetin acetyl hexoside	Flavonoids	Flavonols
Li512112	563.1	4.95	564.088	[M-H]-	$C_{25}H_{24}O_{15}$	Isorhamnetin hexose-malonate	Flavonoids	Flavonols
Li512117	611.16	4.39	610.126	[M+H]+	$C_{27}H_{30}O_{16}$	Quercetin 3-O-rhanosylgalactoside	Flavonoids	Flavonols
N3149	433.08	4.56	434.084	[M-H]-	$C_{20}H_{18}O_{11}$	Quercetin-O-pentoside	Flavonoids	Flavonoid
N3343	489.11	4.76	490.111	[M-H]-	$C_{23}H_{22}O_{12}$	Kaempferol-O-acetylhexoside	Flavonoids	Flavonoid
N3573	535.11	5.06	536.116	[M-H]-	$C_{24}H_{24}O_{14}$	Laricitrin-O-acetylhexoside	Flavonoids	Flavonoid
mws0036	609.19	4.46	610.16	[M-H]-	$C_{28}H_{34}O_{15}$	Hesperidin	Flavonoids	Dihydroflavone
mws0042	305	3.03	306.061	[M-H]-	$C_{15}H_{14}O_7$	(-)-Epigallocatechin	Flavonoids	Flavanols
mws0044	303.06	4.59	304.047	[M-H]-	$C_{15}H_{12}O_7$	Taxifolin	Flavonoids	Dihydroflavonol
mws0045	449.1	4.78	448.082	[M+H]+	$C_{21}H_{20}O_{11}$	Quercitrin	Flavonoids	Flavonols
mws0047	579.17	4.82	578.137	[M+H]+	$C_{27}H_{30}O_{14}$	Rhoifolin	Flavonoids	Flavonoid
mws0049	307	2.84	306.061	[M+H]+	$C_{15}H_{14}O_7$	(+)-Gallocatechin	Flavonoids	Flavanols
mws0054	289.08	3.49	290.067	[M-H]-	$C_{15}H_{14}O_6$	Catechin	Flavonoids	Flavanols
mws0055	373.12	6.5	372.105	[M+H]+	$C_{20}H_{20}O_7$	Tangeretin	Flavonoids	Flavonols
mws0056	463.1	4.14	464.076	[M-H]-	$C_{21}H_{20}O_{12}$	Myricitrin	Flavonoids	Flavonols
mws0058	299.06	6.1	300.053	[M-H]-	$C_{16}H_{12}O_6$	Diosmetin	Flavonoids	Flavonoid

mws0059	609.1	4.23	610.126	[M-H]-	C27H30O16	Rutin	Flavonoids	Flavonols
mws0061	463.1	4.15	464.076	[M-H]-	C21H20O12	Hyperin	Flavonoids	Flavonols
mws0064	287.06	5.53	288.053	[M-H]-	C15H12O6	Eriodictyol	Flavonoids	Dihydroflavone
mws0066	315.06	6.31	316.047	[M-H]-	C16H12O7	Isorhamnetin	Flavonoids	Flavonols
mws0089	447.09	4.65	448.082	[M-H]-	C21H20O11	Kaempferol 7-O-glucosdie	Flavonoids	Flavonols
mws0091	463.09	4.48	464.076	[M-H]-	C21H20O12	Isoquercitrin	Flavonoids	Flavonols
mws0169	595	3.1	630.107	[M-Cl]+	C27H31ClO15	Cyanidin 3-rutinoside(Keracyanin chloride)	Flavonoids	Anthocyanins
mws0183	153.1	3.05	154.021	[M-H]-	C7H6O4	Protocatechuic acid	Flavonoids	Flavanols
mws0463	301.08	6.13	302.067	[M-H]-	C16H14O6	Hesperetin	Flavonoids	Dihydroflavone
mws0789	255.1	6.03	256.064	[M-H]-	C15H12O4	Pinocembrin(Dihydrochrysin)	Flavonoids	Dihydroflavone
mws0791	593	5.4	594.166	[M-H]-	C28H34O14	Poncirin(Isosakuranetin-7-neohesperidoside)	Flavonoids	Dihydroflavone
mws0836	577.3	3.16	578.12	[M-H]-	C30H26O12	Procyanidin B1	Tannins	Proanthocyanidins
mws0856	463	4.3	464.076	[M-H]-	C21H20O12	Spiraeoside	Flavonoids	Flavonols
mws0914	271	5.95	272.058	[M-H]-	C15H12O5	Pinobanksin	Flavonoids	Dihydroflavonol
mws0919	431	4.95	432.088	[M-H]-	C21H20O10	Kaempferin	Flavonoids	Flavonols
mws0920	301	4.88	302.033	[M-H]-	C15H10O7	Tricetin	Flavonoids	Flavonoid
mws1000	287	4.07	288.053	[M-H]-	C15H12O6	Fustin	Flavonoids	Dihydroflavonol
mws1002	345	6.09	346.056	[M-H]-	C17H14O8	Syringetin	Flavonoids	Flavonols
mws1048	449.1	3.18	448.082	[M+H]+	C21H20O11	Cyanidin 3-O-galactoside	Flavonoids	Anthocyanins
mws1066	579.2	4.55	580.152	[M-H]-	C27H32O14	Narirutin	Flavonoids	Dihydroflavone
mws1068	285.05	6.18	286.038	[M-H]-	C15H10O6	Kaempferol	Flavonoids	Flavonols
mws1073	595.2	3.68	594.132	[M+H]+	C27H30O15	Apigenin 6,8-C-diglucoside	Flavonoids	Flavonoid
mws1094	287.06	5.08	288.053	[M-H]-	C15H12O6	Dihydrokaempferol	Flavonoids	Dihydroflavonol
mws1140	271.07	6.16	272.058	[M-H]-	C15H12O5	Naringenin chalcone(4,2',4',6'-	Flavonoids	Chalcones

						Tetrahydroxychalcone)		
mws1174	312.9	6.13	314.067	[M-H]-	C17H14O6	3-O-Acetylpinobanksin	Flavonoids	Dihydroflavonol
mws1179	433	4.79	434.102	[M-H]-	C21H22O10	Naringenin-7-O-glucoside	Flavonoids	Dihydroflavone
mws1290	593.14	5.42	594.114	[M-H]-	C30H26O13	Tiliroside	Flavonoids	Flavonols
mws1329	463.09	4.5	464.076	[M-H]-	C21H20O12	Gossypitrin	Flavonoids	Flavonols
mws1361	449.1	4.47	450.096	[M-H]-	C21H22O11	Astilbin	Flavonoids	Dihydroflavonol
mws1434	431.11	4.2	432.088	[M-H]-	C21H20O10	Isovitexin	Flavonoids	Flavonoid carbonoside
mws1454	477.1	5.04	478.125	[M-H]-	C23H26O11	Persicoside	Flavonoids	Dihydroflavone
mws1519	595.17	4.2	596.146	[M-H]-	C27H32O15	Eriocitrin	Flavonoids	Dihydroflavone
mws1661	609.18	4.65	608.146	[M+H]+	C28H32O15	Diosmin	Flavonoids	Flavonoid
mws2209	447.1	4.66	448.082	[M-H]-	C21H20O11	Astragalin	Flavonoids	Flavonols
mws2471	303.05	4.79	303.04	[M]+	C15H11O7	Delphinidin	Flavonoids	Anthocyanins
mws4183	433.08	4.68	434.068	[M-H]-	C20H18O11	Quercetin-3-O-α-L-arabinopyranoside(guaijaverin)	Flavonoids	Flavonols
pmb0563	301.1	4.48	301.06	[M]+	C16H13O6	Peonidin	Flavonoids	Anthocyanins
pmb0565	509.1	3.86	508.1	[M+H]+	C23H24O13	Syringetin 3-O-hexoside	Flavonoids	Flavonols
pmb0592	771.1	4.26	770.19	[M+H]+	C34H42O20	Chrysoeriol O-hexosyl-O-rutinoside	Flavonoids	Flavonoid
pmb0603	625.1	4.62	624.14	[M+H]+	C28H32O16	Chrysoeriol O-hexosyl-O-hexoside	Flavonoids	Flavonoid
pmb0608	549.1	5.02	548.094	[M+H]+	C25H24O14	Chrysoeriol O-malonylhexoside	Flavonoids	Flavonoid
pmb0618	627.1	3.26	626.155	[M+H]+	C28H34O16	8-C-Hexosyl-hesperetin O-hexoside	Flavonoids	Flavonoid carbonoside
pmb0620	787.1	3.28	786.184	[M+H]+	C34H42O21	Chrysoeriol 6-C-hexoside 8-C-hexoside-O-hexoside	Flavonoids	Flavonoid
pmb0645	627.1	3.87	626.155	[M+H]+	C28H34O16	6-C-Hexosyl-hesperetin O-hexoside	Flavonoids	Flavonoid carbonoside

pmb0663	611.1	4.21	610.153	[M+H]+	C27H30O16	8-C-Hexosyl-luteolin O-hexoside	Flavonoids	Flavonoid carbonoside
pmb0665	611.2	4.17	610.126	[M+H]+	C27H30O16	Luteolin 8-C-hexosyl-O-hexoside	Flavonoids	Flavonoid
pmb0706	713.1	3.43	712.118	[M+H]+	C30H32O20	Quercetin 5-O-malonylhexosyl-hexoside	Flavonoids	Flavonoid
pmb0709	713.1	3.97	712.118	[M+H]+	C30H32O20	Quercetin 7-O-malonylhexosyl-hexoside	Flavonoids	Flavonoid
pmb2947	865.1	4.01	866.172	[M-H]-	C45H38O18	Catechin-catechin-catechin	Tannins	Proanthocyanidins
pmb2957	465.1	3	466.073	[M-H]-	C24H18O10	Cyanidin O-syringic acid	Flavonoids	Anthocyanins
pmb2979	549.2	4.49	550.108	[M-H]-	C25H26O14	Hesperetin O-malonylhexoside	Flavonoids	Dihydroflavonol
pmb2991	739.1	4.22	740.181	[M-H]-	C33H40O19	Apigenin O-hexosyl-O-rutinoside	Flavonoids	Flavonoid
pmb2999	461.1	4.3	462.096	[M-H]-	C22H22O11	Chrysoeriol 5-O-hexoside	Flavonoids	Flavonoid
pmb3000	503.1	4.44	504.105	[M-H]-	C24H24O12	Chrysoeriol O-acetylhexoside	Flavonoids	Flavonoid
pmb3002	607.1	4.49	608.146	[M-H]-	C28H32O15	Chrysoeriol 7-O-rutinoside	Flavonoids	Flavonoid
pmb3006	431.1	4.61	432.088	[M-H]-	C21H20O10	Apigenin 7-O-glucoside(Cosmosiin)	Flavonoids	Flavonoid
pmb3013	519.1	4.78	520.1	[M-H]-	C24H24O13	Isorhamnetin O-acetyl-hexoside	Flavonoids	Flavonols
pmb3023	449.1	3.8	450.096	[M-H]-	C21H22O11	Eriodictyol C-hexoside	Flavonoids	Dihydroflavone
pmb3026	505.1	4.41	506.085	[M-H]-	C23H22O13	Quercetin O-acetylhexoside	Flavonoids	Flavonoid
pmb3114	561.1	3.95	562.125	[M-H]-	C30H26O11	Epicatechin-epiafzelechin	Tannins	Proanthocyanidins
pmb3894	329.1	6.36	330.061	[M-H]-	C17H14O7	Di-O-methylquercetin	Flavonoids	Flavonols
pme0001	609.2	4.53	610.16	[M-H]-	C28H34O15	Hesperetin 7-O-neohesperidoside(Neohesperidin)	Flavonoids	Dihydroflavone
pme0088	285	5.54	286.038	[M-H]-	C15H10O6	Luteolin	Flavonoids	Flavonoid
pme0321	431.11	5.22	432.088	[M-H]-	C21H20O10	Kaempferol 7-O-rhamnoside	Flavonoids	Flavonols
pme0368	579.16	4.46	578.137	[M+H]+	C27H30O14	Apigenin 7-rutinoside(Isorhoifolin)	Flavonoids	Flavonoid
pme0369	593.16	4.29	594.132	[M-H]-	C27H30O15	Kaempferol 3-O-	Flavonoids	Flavonols

						rutinoside(Nicotiflorin)		
pme0376	271.07	6.04	272.058	[M-H]-	C15H12O5	Naringenin	Flavonoids	Dihydroflavone
pme0434	577	3.37	578.12	[M-H]-	C30H26O12	Procyanidin B2	Tannins	Proanthocyanidins
pme0436	577.1	3.37	578.12	[M-H]-	C30H26O12	Procyanidin B3	Tannins	Proanthocyanidins
pme0460	291	3.77	290.067	[M+H]+	C15H14O6	L-Epicatechin	Flavonoids	Flavanols
pme1201	273.08	6.02	274.073	[M-H]-	C15H14O5	Phloretin	Flavonoids	Chalcones
pme1540	625.17	4.04	624.14	[M+H]+	C28H32O16	Isorhamnetin 3-O-neohesperidoside	Flavonoids	Flavonols
pme1598	463.13	4.31	464.111	[M-H]-	C22H24O11	Hesperetin 5-O-glucoside	Flavonoids	Dihydroflavonol
pme1605	593.16	4.16	594.132	[M-H]-	C27H30O15	Kaempferol 3-O-robinobioside(Biorobin)	Flavonoids	Flavonols
pme1611	433.12	4.22	434.102	[M-H]-	C21H22O10	Isohemiphloin	Flavonoids	Flavonoid carbonoside
pme2459	449.1	4.27	448.082	[M+H]+	C21H20O11	Luteolin 7-O-glucoside(Cynaroside)	Flavonoids	Flavonoid
pme2493	579.2	4.27	578.137	[M+H]+	C27H30O14	Kaempferol 3,7-dirhamnoside(Kaempferitrin)	Flavonoids	Flavonols
pme2954	303.04	5.58	302.033	[M+H]+	C15H10O7	Quercetin	Flavonoids	Flavonols
pme3211	463	4.31	464.076	[M-H]-	C21H20O12	Quercetin 3-O-glucoside(Isotrifoliin)	Flavonoids	Flavonols
pme3475	273.07	6.1	272.058	[M+H]+	C15H12O5	Butin	Flavonoids	Dihydroflavone
pmf0371	315.05	5.22	316.047	[M-H]-	C16H12O7	Syringaldehyde	Flavonoids	Flavonoid
pmf0374	477.1	4.69	478.091	[M-H]-	C22H22O12	2,6-Dimethyl-7-octene-2,3,6-triol	Flavonoids	Flavonoid
pmf0392	315.09	6.15	316.081	[M-H]-	C17H16O6	Apigeninidin chloride	Flavonoids	Flavonoid
pmn001415	451.1	4.45	452.093	[M-H]-	C24H20O9	Catechin-(7,8-bc)-4β-(3,4-dihydroxyphenyl)-dihydro-2-(3H)-pyranone	Flavonoids	Flavanols
pmn001416	451.1	4.72	452.093	[M-H]-	C24H20O9	Catechin-(7,8-bc)-4α-(3,4-dihydroxyphenyl)-dihydro-2-(3H)-	Flavonoids	Flavanols

						pyranone		
pmn001583	609.1	4.06	610.126	[M-H]-	C27H30O16	Bioquercetin	Flavonoids	Flavonols
pmn001637	417.1	4.91	418.073	[M-H]-	C20H18O10	Juglanin	Flavonoids	Flavonols
pmn001640	449.1	4.29	450.062	[M-H]-	C20H18O12	Betmidin(Myricetin 3-α-L-arabinofuranoside)	Flavonoids	Flavonols
pmn001641	475.1	4.73	476.076	[M-H]-	C22H20O12	Kaempferide 3-O-β-D-glucuronide	Flavonoids	Flavonols
pmn001644	519.1	4.73	520.065	[M-H]-	C23H20O14	Quercetin 3-O-β-(2''-O-acetylβ-D-glucuronide)	Flavonoids	Flavonols
pmn001645	533.1	4.78	534.08	[M-H]-	C24H22O14	Isorhamnetin 3-O-β-(2''-O-acetyl-β-D-glucuronide)	Flavonoids	Flavonols
pmn001646	865.2	3.82	866.172	[M-H]-	C45H38O18	Procyanidin C1	Tannins	Proanthocyanidins
pmn001647	865.2	3.62	866.172	[M-H]-	C45H38O18	Procyanidin C2	Tannins	Proanthocyanidins
pmn001649	1153.3	3.9	1154.225	[M-H]-	C60H50O24	Cinnamtannin A2	Tannins	Proanthocyanidins
pmn001650	1153.3	3.62	1154.225	[M-H]-	C60H50O24	Cinnamtannin B2	Tannins	Proanthocyanidins
pmn001667	577.1	4.28	578.12	[M-H]-	C30H26O12	Procyanidin B4	Tannins	Proanthocyanidins
pmn001713	593.17	3.65	594.132	[M-H]-	C27H30O15	Luteolin-7-O-β-D-rutinoside	Flavonoids	Flavonoid
pmp000004	331.08	6.4	330.061	[M+H]+	C17H14O7	Jaceosidin	Flavonoids	Anthocyanins
pmp000013	507.15	5.34	506.12	[M+H]+	C24H26O12	Eupatilin 3-glucoside	Flavonoids	Flavonoid
pmp000127	625.2	3.85	624.14	[M+H]+	C28H32O16	Chysoeriol-6,8-di-C-glucoside	Flavonoids	Flavonoid carbonoside
pmp000237	579.13	4.37	578.103	[M+H]+	C26H26O15	Apigenin-6-C-2-glucuronylxyloside	Flavonoids	Flavonoid carbonoside
pmp000241	625.14	3.7	624.106	[M+H]+	C27H28O17	Luteolin-6-C-2-glucuronylglucoside	Flavonoids	Flavonoid
pmp000275	581.16	4.05	580.134	[M+H]+	C30H28O12	Gambiriin A1	Tannins	Proanthocyanidins
pmp000414	579.17	3.35	578.137	[M+H]+	C27H30O14	Puerarin 4'-O-D-glucoside	Flavonoids	Flavonoid carbonoside

pmp000579	463.12	4.87	462.096	[M+H]+	C22H22O11	Diosmetin-7-O-galactoside	Flavonoids	Flavonoid
pmp000589	551.1	4.63	550.074	[M+H]+	C24H22O15	Quercetin-7-O-(6'-O-malonyl)-β-D-glucoside	Flavonoids	Flavonols
pmp000593	595.16	4.26	594.132	[M+H]+	C27H30O15	Luteolin-7-O-rutinoside	Flavonoids	Flavonoid
pmp000594	609.18	4.69	608.146	[M+H]+	C28H32O15	Diosmetin-7-O-rutin	Flavonoids	Flavonoid
pmp000596	627.15	3.37	626.12	[M+H]+	C27H30O17	Quercetin 3,7-bis-O-β-D-glucoside	Flavonoids	Flavonols
pmp001080	609.18	4.62	608.146	[M+H]+	C28H32O15	Neodiosmin	Flavonoids	Flavonoid
pmp001106	595.17	3.61	594.132	[M+H]+	C27H30O15	Vitexin-2-O-D-glucopyranoside	Flavonoids	Flavonoid carbonoside
pmp001288	593.2	5.29	592.152	[M+H]+	C28H32O14	Linarin	Flavonoids	Flavonoid
pmp001309	465.1	4.15	464.076	[M+H]+	C21H20O12	6-Hydroxykaempferol-7-O-glucoside	Flavonoids	Flavonols
pmp001310	627.2	3.82	626.12	[M+H]+	C27H30O17	6-Hydroxykaempferol-3,6-O-Diglucoside	Flavonoids	Flavonols
pmp001311	627.2	3.38	626.12	[M+H]+	C27H30O17	6-Hydroxykaempferol-7,6-O-Diglucoside	Flavonoids	Flavonols
pmp001312	789.2	3.13	788.164	[M+H]+	C33H40O22	6-Hydroxykaempferol-3,7,6-O-triglycoside	Flavonoids	Flavonols
pmp001314	773.2	3.74	772.17	[M+H]+	C33H40O21	6-Hydroxykaempferol-3-O-rutin-6-O-glucoside	Flavonoids	Flavonols

Table S2. Transcriptome sequencing results.

Sample	Raw Reads	Clean Reads	Clean Base (G)	Error Rate (%)	Q20 (%)	Q30 (%)	GC Content(%)
A1	46882572	45487418	6.82	0.02	96.26	90.71	43.53
A2	49680796	48362748	7.25	0.02	96.75	91.6	43.46
A3	44018268	42750648	6.41	0.02	96.41	91.02	43.61
B1	53532554	52039998	7.81	0.02	96.34	90.86	43.53
B2	50140368	48727742	7.31	0.02	96.42	91.03	43.65
B3	42530114	41323750	6.2	0.02	96.37	90.93	43.82
C1	46728072	45510164	6.83	0.02	96.55	91.22	43.63
C2	45289608	43888338	6.58	0.02	96.8	91.73	43.73
C3	47733854	46543078	6.98	0.02	96.53	91.13	43.57
D1	45572000	44360742	6.65	0.02	96.68	91.49	43.8
D2	46561416	45160240	6.77	0.02	96.54	91.15	43.74
D3	45964540	44737480	6.71	0.02	96.88	91.89	43.89

Table S3. Statistics of transcription splicing results.

Type	Number	Mean Length	N50	N90	Total Bases
Transcript	378253	807	1169	355	305279552
Unigene	327752	889	1228	415	291421747

Table S4. Gene function annotation.

Database	Number of Genes	Percentage (%)
KEGG	162931	49.71
NR	215465	65.74
SwissProt	147661	45.05
Trembl	213750	65.22
KOG	126865	38.71
GO	173383	52.9
Pfam	143521	43.79
Annotated in at least one Database	220800	67.37
Total Unigenes	327752	100

Table S5. Primers in this study.

Primers	Sequence (5'-3')	Description
ZbCHI-F	CGAGCTCATGGGTTTCATGGACTTTGA	Gene amplification
ZbCHI-R	ACGTCGACGCACCTCGTTACCAAACTAG	Gene amplification
ZbFLS-F	CGAGCTCATGGTGGAAGTGGCTGAGAG	Gene amplification
ZbFLS-R	ACGTCGACTTGATGGGTGACTAATAAAA	Gene amplification
ZbANR-F	CGAGCTCATGGCAAGCCAATTCACCCA	Gene amplification
ZbANR-R	ACGTCGACCGTTGGAACATTGTACTCCGG	Gene amplification
pBI121-ZbCHI-F	AGAACACGGGGGACTCTAGAGGATCCATGGGTTTCATGGACTTTGA	Transient expression
pBI121-ZbCHI-R	TTGAACGATCGGGGAAATTCGAGCTCTTAGCACCTCGTTACCAAAC	Transient expression
pBI121-ZbFLS-F	AGAACACGGGGGACTCTAGAGGATCCATGGTGGAAGTGGCTGAGAG	Transient expression
pBI121-ZbFLS-R	TTGAACGATCGGGGAAATTCGAGCTCTTATTGATGGGTGACTAATA	Transient expression
pBI121-ZbANR-F	AGAACACGGGGGACTCTAGAGGATCCATGGCAAGCCAATTCACCCA	Transient expression
pBI121-ZbANR-R	TTGAACGATCGGGGAAATTCGAGCTCTCACGTTGGAACATTGTACTC	Transient expression
2799.119461-F	GTGGGTGCTGGCAGGACAAAG	RT-qPCR
2799.119461-R	CAACAGGACACTCGCACACTCTTC	RT-qPCR
2799.115450-F	GGGGTTAGGTGTAGAAGGG	RT-qPCR
2799.115450-F	AAGCCAGGGACGTGATTAG	RT-qPCR
2799.111612-F	GCCTGATATTCCTAGCAGCGTAGC	RT-qPCR
2799.111612-R	ATATGGGCACGGCAAACATCCTC	RT-qPCR
2799.191516-F	CCGCACCAAACGGTAACAG	RT-qPCR
2799.191516-R	GCCCCACAAAGCCAACACA	RT-qPCR
2799.191577-F	GTTGTTGCTATGGTTTTGG	RT-qPCR
2799.191577-R	TTGATTGCCTCTTGTTCTT	RT-qPCR
2799.191506-F	TTCTAAAATGAACACAAGC	RT-qPCR

2799.191506-R	AAGACAAACCTCCAACCAC	RT-qPCR
2799.191476-F	CTGCTGCCAATGAAAATAC	RT-qPCR
2799.191476-R	AATCACGCCGCTTCCTACT	RT-qPCR
2799.160752-F	GACCGAGCCAGCTCAAGCAATG	RT-qPCR
2799.160752-R	ACGTCCTCACCTCCTGCCTTATC	RT-qPCR
2799.146229-F	ACAAGCCTCTTCATGCCTCTGTTG	RT-qPCR
2799.146229-R	AGCCTCCTCCTCTTCCTGTTGC	RT-qPCR
2799.242322-F	TTTGGGTGGTGGTTGTCG	RT-qPCR
2799.242322-R	TCTCCCCCCTCGGTCGGT	RT-qPCR
UBA -F	GACTTAGGGGAGGGATTATTGAG	RT-qPCR
UBA -R	TTCTTCTTCCGACAGTTTACAGC	RT-qPCR
TIF -F	TTCCTCCCATTACGTTGCT	RT-qPCR
TIF -R	GCTGGTTACGGACTCTTTG	RT-qPCR
